# Coordinate regulation of methanol utilization pathway genes of *Komagataella phaffii* by transcription factors and chromatin modifiers

**DOI:** 10.3389/fmicb.2022.991192

**Published:** 2022-09-06

**Authors:** Aditi Gupta, Pundi N. Rangarajan

**Affiliations:** Department of Biochemistry, Indian Institute of Science, Bangalore, India

**Keywords:** *Komagataella phaffii*, histone acetyltransferase, MXR1, methanol utilization pathway, RNA-Seq, Gcn5, SNF1, Trm1

## Abstract

The methylotrophic yeast *Komagataella phaffii* (*a.k.a. Pichia pastoris*) harbors a methanol utilization (MUT) pathway, enabling it to utilize methanol as the sole source of carbon. The nexus between transcription factors such as Mxr1p and Trm1p and chromatin-modifying enzymes in the regulation of genes of MUT pathway has not been well studied in *K. phaffii*. Using transcriptomics, we demonstrate that Gcn5, a histone acetyltransferase, and Gal83, one of the beta subunits of nuclear-localized SNF1 (sucrose non-fermenting 1) kinase complex are essential for the transcriptional regulation by the zinc finger transcription factors Mxr1p and Trm1p. We conclude that interactions among Gcn5, Snf1, Mxr1p, and Trm1p play a critical role in the transcriptional regulation of genes of MUT pathway of *K. phaffii*.

## Introduction

In eukaryotes, the binding of transcription factors to the promoter region of target genes in a sequential manner leads to their activation or repression through the recruitment of co-regulators. Co-regulatory proteins often contain the enzymatic activities necessary for chromatin modification to facilitate or restrict access to the underlying DNA. For example, histone acetyltransferases (HATs) interact with a wide spectrum of transcription factors and other regulatory proteins to affect gene expression (Hermanson et al., 2002). Co-activators such as the mediator complex comprise TATA-box binding protein-associated factors-TAFII250 and TFIIIC, bind to transcription factors, recruit RNA polymerase II and interact with the general transcription apparatus (Malik and Roeder, 2000; Sterner and Berger, 2000). Gene expression can also be modulated by DNA unwinding proteins as evident from the ATP-dependent DNA unwinding activities of yeast SWI/SNF family proteins (Lemon et al., 2001).

The methylotrophic yeast, *Komagataella phaffii* (*a.k.a. Pichia pastoris*) harbors a methanol utilization (MUT) pathway, the key enzymes of which are methanol-inducible (Cregg et al., 1989). Alcohol oxidase 1 (AOX1), the first enzyme of the MUT pathway, is the most abundant enzyme in cells metabolizing methanol, and its promoter is widely used for methanol-inducible expression of heterologous genes (Cereghino and Cregg, 2000). Several transcription factors such as Mxr1p, Trm1p/Prm1p, Mit1p, Rop1p, Mig1p, Mig2p, and Nrg1p regulate the transcription of *AOXI* and other genes of MUT pathway (Lin-Cereghino et al., 2006; Kumar and Rangarajan, 2011; Sahu et al., 2014; Wang et al., 2016a,b; Shi et al., 2018). In addition to methanol, *K. phaffii* can also metabolize a diverse range of carbon compounds such as glucose, glycerol, ethanol, acetate, fatty acids, and amino acids as the sole source of carbon. Mxr1p functions as a global regulator of several of these metabolic pathways (Sahu and Rangarajan, 2016a,b; Gupta et al., 2021).

The interactions between transcription factors and chromatin modifying enzymes have not been well studied in *K. phaffii*. In this study, we examined the role of General Control Non-repressed 5 protein (Gcn5), a histone acetyltransferase (HAT), and sucrose non-fermenting 1 (Snf1) kinase complex in regulating carbon metabolism of *K. phaffii*. Gcn5, a 55 kDa protein, is one of the first and well-characterized HATs and is the founding member of the GCN5-related N-acetyltransferases (GNAT) family of HATs (Sterner and Berger, 2000). Gcn5 preferentially acetylates lysine residues in histones H3 and H2B and functions as the catalytic subunit of the SAGA (Spt-Ada-Gcn5 acetyltransferase) complex (Grant et al., 1997). Snf1 kinase complex is a heterotrimeric protein comprising of a catalytic α-subunit known as Snf1 and a regulatory γ subunit known as Snf4 as well as a β subunit that anchors the subunit to the γ subunit and could be any one of three – Gal83, Sip1, and Sip2. Snf1 and Snf4 were identified in the screening of sucrose utilization defective mutants (Schüller, 2003; Coccetti et al., 2018). Snf1 is the yeast homolog of the AMP-activated protein kinase of higher eukaryotes and is often referred to as the “energy sensor of the cell” (Hardie et al., 1998). In *S. cerevisiae*, Gcn5 and Snf1 collaborate with transcription factors such as Adr1p and Cat8 in regulating the expression of genes involved in the metabolism of non-fermentable carbon sources (Abate et al., 2012). There are no reports on the role of Gcn5 and Snf1 in the regulation of carbon metabolism of *K. phaffii* except for a study by Shen et al. (2016) wherein deletion of *GAL83* was shown to cause downregulation of *AOXI* mRNA resulting in growth retardation. Here, we investigated the potential cross-talk in the gene regulatory activities of Gcn5, Snf1, and the zinc finger transcription factors Mxr1p and Trm1p. We demonstrate that Mxr1p and Trm1p are unable to activate the transcription of key genes of the MUT pathway in Δ*gcn5* and Δ*gal83*. *Komagataella phaffii Δgcn5* and Δ*gal83* are defective in metabolizing carbon compounds such as acetate, ethanol, and methanol. We have identified genes of methanol metabolism that are differentially expressed in Δ*gcn5* and Δ*gal83* as well as Δ*mxr1, and Δtrm1* by high-throughput RNA-Seq. Comparative analysis of transcriptomes led to the identification of several genes that are coordinately controlled by multiple regulators.

## Materials and methods

### Growth media and culture conditions

*Komagataella phaffii* cells were maintained on plates containing 1% yeast extract, 2% peptone, 2% dextrose (YPD), and 2% agar. A single colony was grown overnight in YPD at 30°C in an orbital shaker at 180 rpm, washed twice with sterile distilled water followed by transfer to desired media consisting of YP (1% yeast extract, 2% peptone) and an appropriate carbon source (2%) such as dextrose, acetate, methanol, or ethanol. Minimal media consisted of 0.67% yeast nitrogen base (YNB) with ammonium sulfate and without histidine together with 1% methanol (YNBM), supplemented with L-histidine (20 μg/ml). Yeast transformations were performed by electroporation (Gene Pulsar, Bio-Rad). Yeast extract and peptone were procured from the same vendors as mentioned previously (Gupta and Rangarajan, 2022).

### Growth kinetics

A single colony of yeast cells grown till late log phase was pelleted and washed twice with autoclaved Milli-Q™ water under sterile conditions and resuspended in sterile water. These cells were inoculated into specific media at an initial A_600_ of 0.06–0.1 and grown at 30°C at 180 rpm in an orbital shaker. A_600_ was measured at regular intervals till cells attained the stationary phase.

### Quantitative real-time PCR

*Komagataella phaffii* cells were cultured overnight in YPD, centrifuged, the entire cell mass was transferred to a medium containing methanol, cultured for 6 h to allow methanol-inducible transactivation or repression of genes, and RNA was isolated using RNA isolation kit (Cat. # Z3100, Promega) according to manufacturer’s instructions. cDNA prepared from DNase-treated RNA was used for carrying out qPCR as described previously (Gupta et al., 2021). ΔΔCt method was used for calculating the relative mRNA expression levels, wherein the ΔCt value of a sample, which is the Ct value of a gene relative to that of 18S rRNA, is normalized to that of the control.

### RNA-sequencing and data analysis

*Komagataella phaffii* strains *GS115, Δmxr1, Δgal83,* and *Δgcn5* were cultured in duplicates, along with a single replicate of *Δtrm1* in YNBM for 14 h followed by total RNA preparation using Qiagen RNeasy kit according to the manufacturer’s protocol. Sequencing was performed at Clevergene, Bangalore, using Illumina HiSeq. QC passed reads were aligned onto indexed *K. phaffii* CBS 7435 reference genome (GCA_900235035.1_ASM90023503v1) by Bowtie2 aligner. On average, 94.73% of the reads could be mapped to the reference genome. Feature counts software gave gene level expression values as read counts and the spearman correlation along with principal component analysis gave the similarity between biological replicates. Differentially expressed genes were analyzed using DESeq2 v1.20 which estimates the variance by treating all samples as if they were replicates of the same condition. The protein sequences were annotated against the Uniref100 protein database using BLASTp module of Diamond. Genes with absolute log2 fold change ≥1.5 and adjusted *p*-value < 0.05 were considered significant. The expression profile of differentially expressed genes across the samples is presented in volcano plots and heatmaps. The transcriptome datasets generated during the current study are available in the GEO DataSets, NCBI, under the accession number GSE186300.

### Generation of *Komagataella phaffii Δgcn5*

*Komagataella phaffii,* Gcn5 (*PAS_chr3_1128,* XP_002493374) is a 448 amino acid protein, annotated as HAT which acetylates N-terminal lysine on histones H2B and H3. It shares 76% amino acid sequence identity with the *S. cerevisiae* Gcn5 (*YGR252W*). *Komagataella phaffii Δgcn5* was generated by replacing the *GCN5* coding region with the zeocin resistance expression cassette, which comprises the zeocin coding sequence flanked by sequence 1 kb upstream of the gene (promoter sequence), and sequence 1 kb downstream of the stop codon of *GCN5* (terminator sequence). In the first step, promoter, zeocin, and terminator regions were amplified. The promoter sequence was amplified using the following primer pair-F1 5′-GCTGGTATGCTCATTGTAAGCCAACG-3′, 848–823 bp upstream of ATG of the gene, and R1 5′-GCTATGGTGTGTGGGGGATCCGCTTGGACTAACGCACTACCATAATTGAAG AACC-3′, which is the reverse complement of 122–91 bp upstream of ATG of the gene and 1–23 bp of the zeocin cassette. Zeocin sequence was amplified using primer pairs F2 5′-GGTTCTTCAATTATGGTAGTGCGTTAGTCCAAGCGGATCCCCCACACACCATAGC-3′, which is 122–91 bp upstream of ATG of the gene and 1–23 bp of zeocin gene and R2 5′-CGAAGAAAGCGAGCAGATATAGTTTGTGGTGCTCACATGTTGGTCTCCAGCTTG-3′, which is reverse complement of 1175–1199 bp of zeocin followed by 41–69 bp downstream of the stop codon in the terminator region of *GCN5.* The terminator was amplified using primer pairs F3 5′-CAAGCTGGAGACCAACATGTGAGCACCACAAACTATATCTGCTCGCTT TCTTCG-3′, which is 1175–1199 bp of zeocin followed by 41–69 bp downstream of stop codon in the terminator region of *GCN5* and R3 5′-CGCCGAAGTACTAAAGGCCATGC-3′, which is reverse complement of 927–949 bp downstream of stop codon in the terminator region of *GCN5*. Finally, these three PCR products were used as templates for the overlapping PCR using primers F1 and R3. This cassette was transformed in *GS115,* and screened by plating on zeocin-resistant plates, followed by PCR and qPCR of genomic DNA and RNA using *KpGCN5*-specific primers, respectively, for strain confirmation.

### Generation of *Komagataella phaffii Δgal83*

*Komagataella phaffii* Gal83 (*PAS_chr1-4_0498,* XP_002490632) is a 422 amino acid protein, annotated as one of the three possible beta-subunits of Snf1 kinase complex. It shares a 46% amino acid sequence identity with *S. cerevisiae* Gal83 (*YER027C*). *Komagataella phaffii Δgal83* was generated by replacing the *GAL83* coding region with the zeocin resistance expression cassette, which comprises the zeocin coding sequence flanked by sequence 1 kb upstream of gene (promoter sequence), and sequence 1 kb downstream of stop codon of *GAL83* (terminator sequence). In the first step, promoter, zeocin, and terminator regions were amplified. The promoter sequence was amplified using the following primer pair-F1 5′-GCATAAGTGAACCTGGGGAGATGATAAGTG-3′, 1,000–971 bp upstream of ATG of the gene, and R1 5′-GCTATGGTGTGTGGGGGATCCGCTGCGGGCGGTTTCGATAAGAAA TGGGTC-3′, which is the reverse complement of 56–30 bp upstream of ATG of the gene and 1–23 bp of the zeocin cassette. Zeocin sequence was amplified using primer pairs F2 5′-GACCCATTTCTTATCGAAACCGCCCGCAGCGGATCCCCCACACACCATAGC-3′, which is 56–30 bp upstream of ATG of the gene and 1–23 bp of zeocin gene and R2 5′-GCTAGGGGCTCGCTCTATGGAAGATTTGCTCACATGTTGGTCTCCAGCTTG-3′, which is reverse complement of 1,175–1,199 bp of zeocin followed by 18–43 bp downstream of stop codon in the terminator region of *GAL83.* The terminator was amplified using primer pairs F3 5′-CAAGCTGGAGACCAACATGTGAGCAAATCTTCCATAGAGCGAGCCCCTAGC-3′, which is 1,175–1,199 bp of zeocin followed by 18–43 bp downstream of stop codon in the terminator region of *GAL83* and R3 5′-ACTTCGTCAACAGAGACGGGATGATG-3′, which is reverse complement of 950–975 bp downstream of stop codon in the terminator region of *GAL83*. Finally, these three PCR products were used as templates for the overlapping PCR using primers F1 and R3. This cassette was transformed in *GS115,* and screened by plating on zeocin-resistant plates, followed by PCR and qPCR of genomic DNA and RNA using *GAL83*-specific primers, respectively, for strain confirmation.

### Generation of *Komagataella phaffii Δmxr1 and Δtrm1*

*Komagataella phaffii Δmxr1* and *Δtrm1* were generated by disrupting the *MXR1* and *TRM1* coding region, respectively, by the zeocin resistance cassette (*zeo^R^*) in *K. phaffii GS115* and have been previously described (Sahu et al., 2014; Sahu and Rangarajan, 2016b).

## Results and discussion

### Gcn5 and Snf1 are key regulators of methanol metabolism of *Komagataella phaffii*

**Figure 1:**
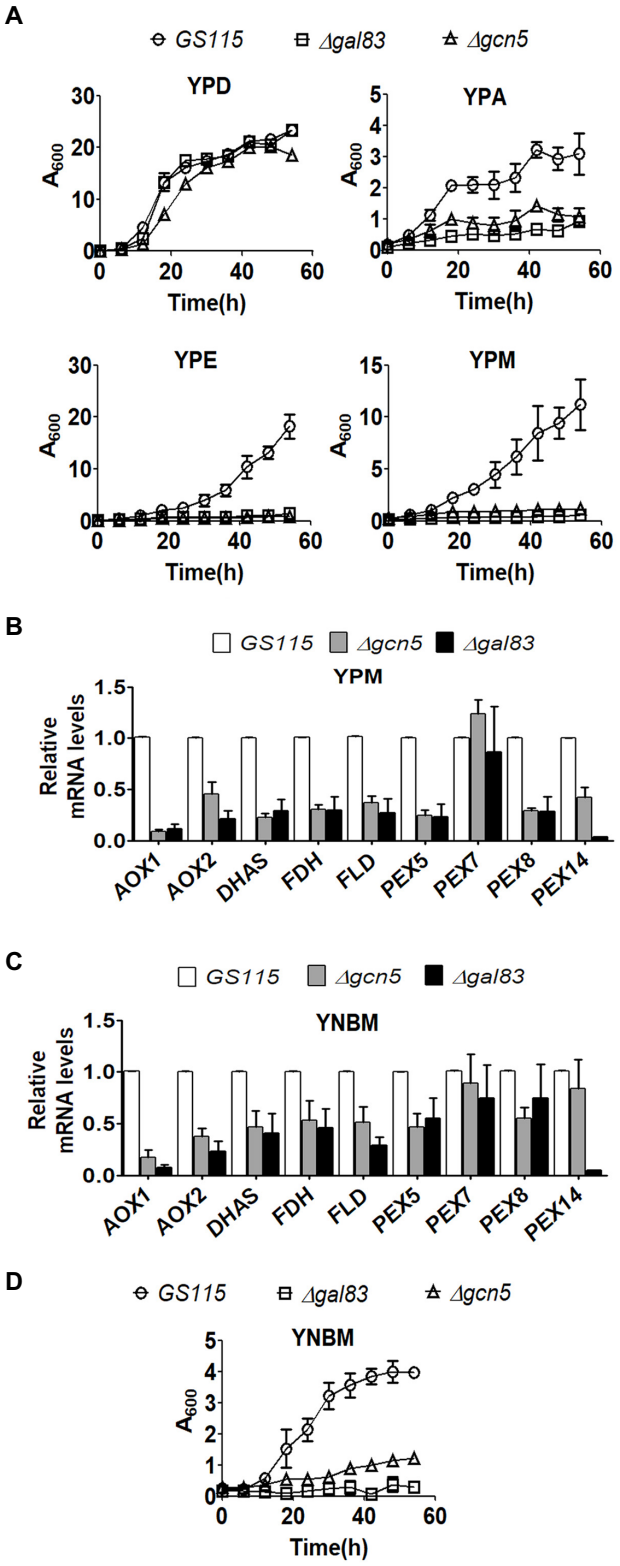
Analysis of the role of Gcn5 and Gal83 in carbon metabolism of *Komagataella phaffii*. **(A)** Growth curves of *GS115, Δgcn5,* and *Δgal83*. Cells were cultured in different media as indicated. Error bars in each figure indicate S.D. *n* = 3. **(B)** Analysis of mRNA levels of key genes of MUT pathway by qPCR in different *K. phaffii* strains cultured in YPM for 6 h. **(C)** Analysis of mRNA levels of key genes of MUT pathway by qPCR in different *K. phaffii* strains cultured in YNBM for 6 h, as indicated. **(D)** Growth curves of *GS115, Δgcn5,* and *Δgal83* cultured in YNBM. Error bars in each figure indicate S.D. *n* = 3.

*Komagataella phaffii* strains used in this study are listed in Table 1. To examine the role of Gcn5 and Snf1 in the regulation of carbon metabolism of *K. phaffii, Δgcn5* and *Δgal83* were generated by replacing the coding regions of *GCN5* and *GAL83* of *K. phaffii GS115* with zeocin resistance (Zeo^R^) expression cassettes *via* homologous recombination (Table 1). In *S. cerevisiae*, Gal83 is one of three alternate beta-subunits of the Snf1 kinase complex that allows nuclear localization of the Snf1 kinase complex in cells cultured in glucose limiting conditions (Coccetti et al., 2018). Thus, the nuclear functions of Snf1 kinase are abrogated in *Δgal83.* Growth of *K. phaffii Δgcn5* and *Δgal83* was examined in nutrient-rich media consisting of 1% yeast extract and 2% peptone (YP) together with an appropriate source of carbon such as 2% glucose (YPD), 2% acetate (YPA), 2% ethanol (YPE), or 2% methanol (YPM). Growth of *Δgcn5* and *Δgal83* was compromised when cultured in YPA, YPE, and YPM but not in YPD (Figure 1A) indicating that Gcn5 and Gal83/Snf1 have an essential role in the regulation of key genes involved in the metabolism of several carbon compounds such as acetate, ethanol, and methanol. In this study, we focused our attention on the regulation of methanol metabolism by Gcn5 and Gal83. In *K. phaffii*, methanol is converted to formaldehyde, dihydroxyacetone-3-phosphate, and finally to formate by the sequential action of alcohol oxidase 1 encoded by *AOX1, AOX2*, dihydroxyacetone phosphate synthase (DHAS), formaldehyde dehydrogenase (FLD), and formate dehydrogenase (FDH) (Yurimoto et al., 2011). The first two reactions of the MUT pathway take place in the peroxisomes, as a result, the genes *PEX5, PEX7,* and *PEX14* encoding the peroxisomal proteins PEX5, PEX7, and PEX14 are also essential for methanol metabolism (van der Klei et al., 2006). Expression of several of these genes is regulated by transcription factors such as Mxr1p, Trm1p, and Mit1p (Lin-Cereghino et al., 2006; Sahu et al., 2014; Wang et al., 2016b). We examined the expression of these genes in *Δgcn5* and *Δgal83* cultured in YPM by qPCR. The results indicate that several genes involved in MUT pathway are significantly downregulated in *Δgcn5* and *Δgal83* cultured in YPM (Figure 1B). Similar results were obtained when *K. phaffii Δgcn5* and *Δgal83* were cultured in a minimal medium containing 0.67% yeast nitrogen base (YNB) and 1% methanol (YNBM; Figure 1C). Growth of *Δgcn5* and *Δgal83* was severely compromised in YNBM as well (Figure 1D). These results indicate that GCN5 and SNF1 are essential for the expression of genes essential for methanol metabolism of *K. phaffii* cultured in YPM as well as in YNBM.

**Table 1:** *Komagataella phaffii* strains used in this study.

**Strain**	**Description**	**References**
*GS115*	*his4*	Lin-Cereghino et al., 2006
*Δmxr1*	*GS115, Ppmxr1Δ::Zeo^r^*	Lin-Cereghino et al., 2006
*Δtrm1*	*GS115, Pptrm1Δ::Zeo^r^*	Sahu et al., 2014
*Δgcn5*	*GS115, Ppgcn5Δ::Zeo^r^*	This study
*Δgal83*	*GS115, Ppgal83Δ::Zeo^r^*	This study

### Analysis of transcriptome of *Δgcn5* and *Δgal83* cultured in YNBM by RNA-Seq

**Figure 2:**
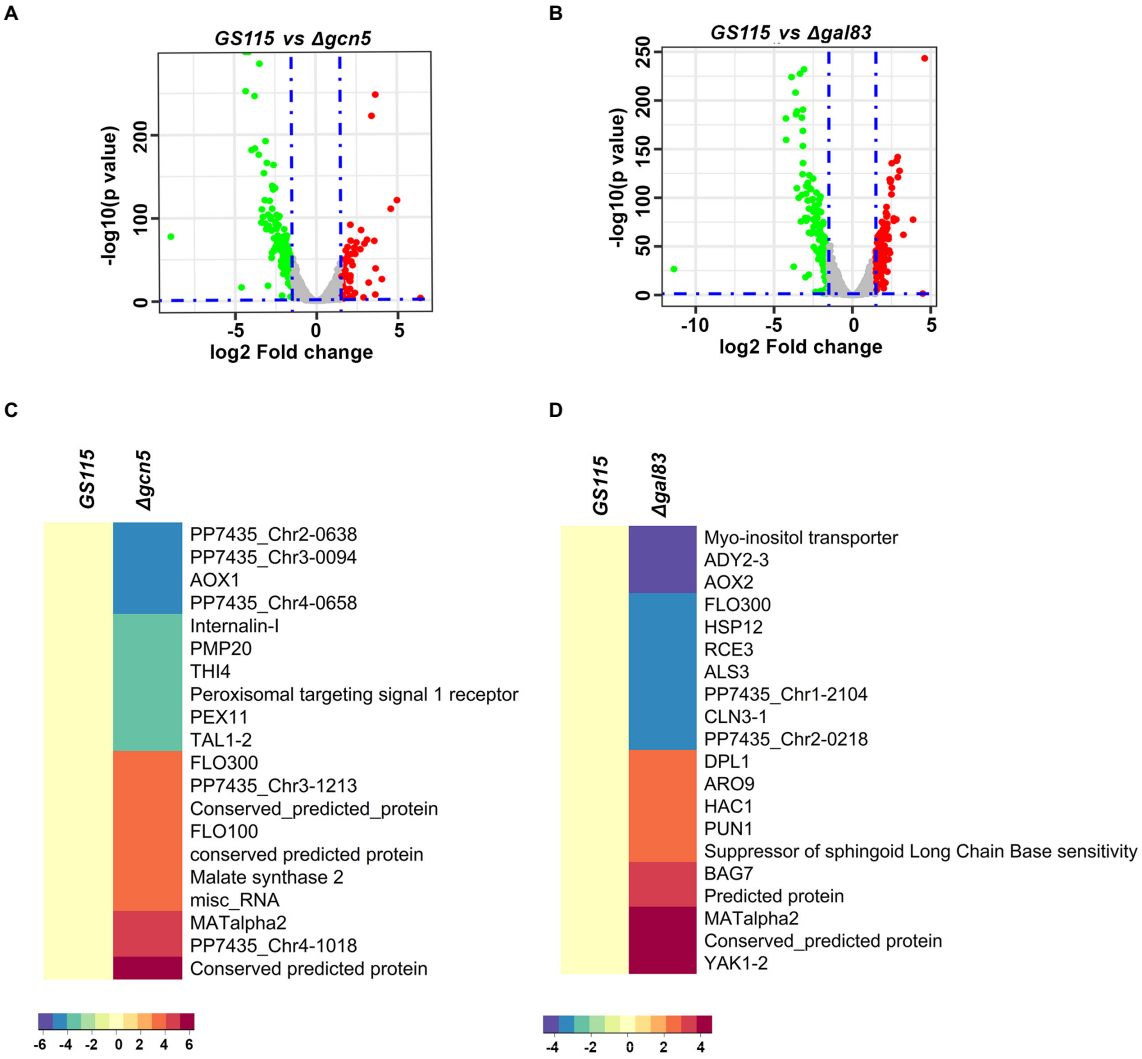
RNA-Seq analysis of *Δgcn5* and *Δgal83* cultured in YNBM. **(A,B)** Volcano plots of *Δgcn5* and *Δgal83* cultured in YNBM depict the overall changes in transcription profile. **(C)** Heat Map of the most highly downregulated and upregulated genes in *Δgcn5* relative to *GS115*. **(D)** Heat map of the most highly downregulated and upregulated genes in *Δgal83* relative to *GS115*.

To identify *K. phaffii* genes whose expression is regulated by Gcn5 and Snf1 during methanol metabolism, RNA-Seq of biological replicates of *GS115, Δgcn5,* and *Δgal83* cultured in YNBM was performed. A total of 249 and 345 genes were found to be differentially regulated with a cut-off of *p*-value < 0.05 and log2foldchange ≥ ±1.5 in *Δgcn5* and *Δgal83,* respectively (Supplementary File 1). Volcano plots for differentially expressed genes are shown in Figures 2A,B. The most highly downregulated and upregulated genes in *Δgcn5* and *Δgal83* are presented as heat maps (Figures 2C,D). It should be noted that *AOXI* is downregulated in *Δgcn5* as well as *Δgal83*. It is 19th among the most downregulated genes in *Δgal83* (Figures 2C,D and Supplementary File 1) and therefore not listed among the top 10 most downregulated genes in *Δgal83* (Figure 2D). Thus, *AOXI* expression is regulated not only by transcriptional activators such as Mxr1p, Mit1p, and Trm1p but also Gcn5 and Snf1. Deletion of Gcn5 and Gal83 also led to upregulation of certain genes suggesting their role in transcriptional repression (Figures 2C,D). Gene ontology analysis of up and downregulated genes in *Δgcn5* and *Δgal83* did not reveal enriched categories.

### Coordinate regulation of genes of methanol metabolism by Mxr1, Trm1, Gcn5, and Snf1

**Figure 3:**
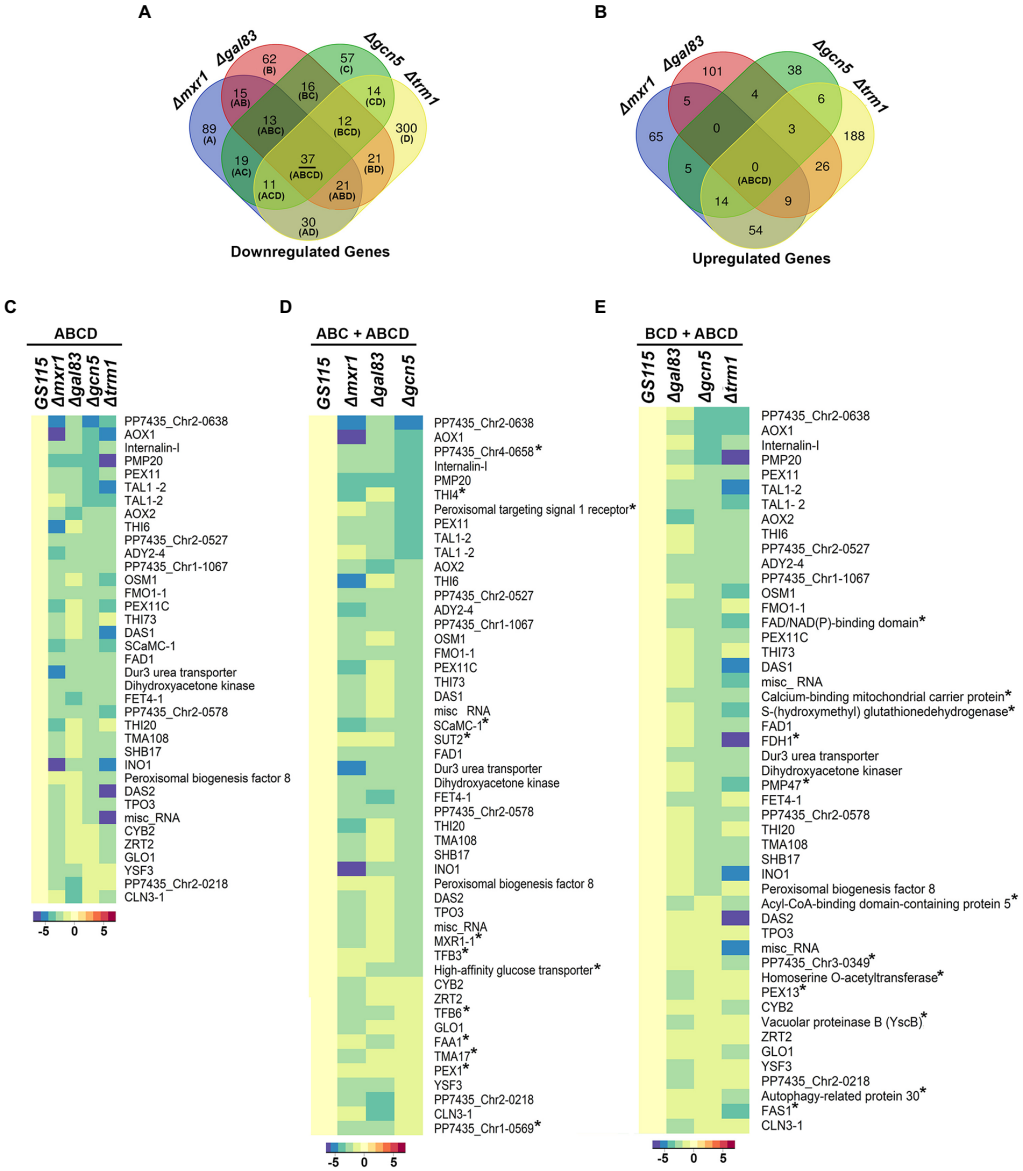
Detailed analysis of differentially expressed genes of *K. phaffii* strains cultured in YNBM. **(A)** Venn diagram of genes downregulated in each *K. phaffii* strain as well as combinations of the four strains. **(B)** Venn diagram of genes upregulated in each *K. phaffii* strain as well as combinations of the four strains. The number in each sector represents differentially expressed genes between the different comparisons. The overlapping number stands for the differentially expressed genes which are common between the different comparisons and the non-overlapping numbers specify the genes unique to each strain. For example, 37 genes (underlined in **A**) are downregulated in *Δmxr1, Δtrm1, Δgcn5* as well as *Δgal83*. Details of genes in each sector are provided in Supplementary File 2. **(C)** Heat map of 37 genes downregulated in *Δtrm1, Δgcn5, Δgal83* as well as *Δmxr1* relative to *GS115* (sector ABCD). **(D)** Heat map of genes downregulated in *Δmxr1, Δgal83,* and *Δgcn5* relative to *GS115* (sectors ABC + ABCD). Genes that are downregulated in *Δmxr1, Δgal83,* and *Δgcn5* but not *Δtrm1* are marked by an asterix. **(E)** Heat map of genes downregulated in *Δgal83*, *Δgcn5,* and *Δtrm1* relative to *GS115* (sectors BCD + ABCD). Genes which are downregulated in *Δtrm1, Δgal83,* and *Δgcn5* but not *Δmxr1* are marked by an asterix.

**Figure 4:**
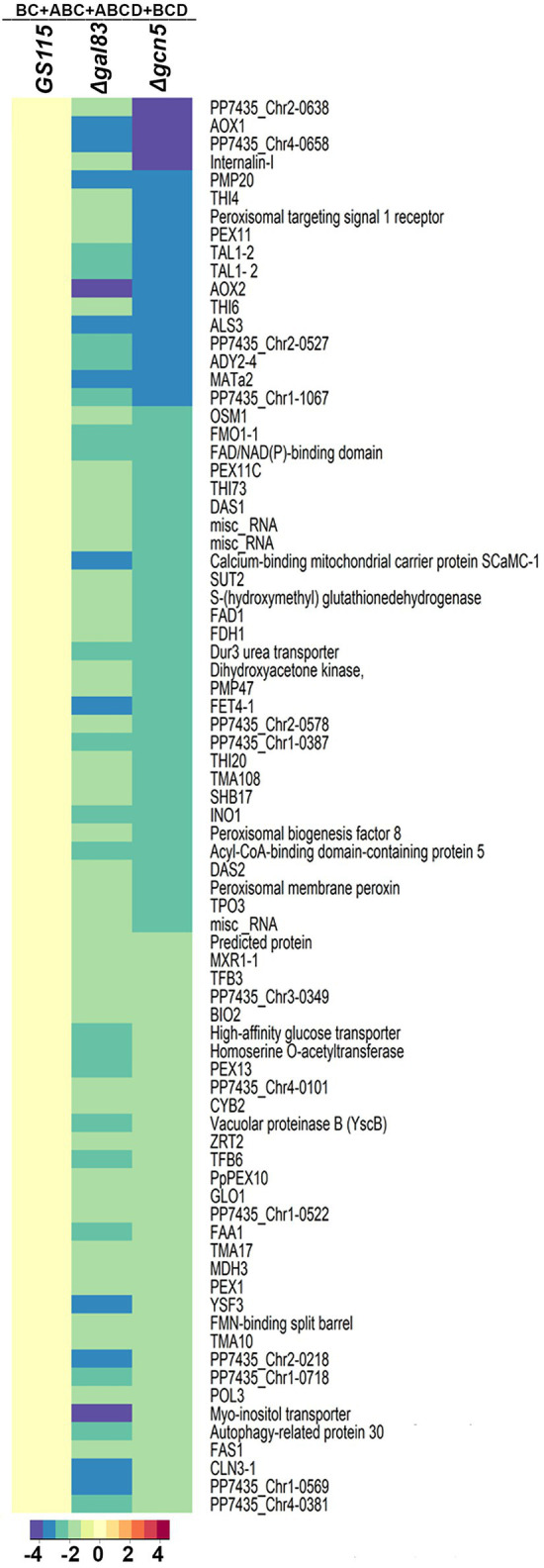
Heat map of genes downregulated in *Δgal83* and *Δgcn5*.

*AOXI, DHAS, FDH,* and *FLD* are known targets of Mxr1p and Trm1p (Lin-Cereghino et al., 2006; Sahu et al., 2014) and their downregulation in *Δgcn5* and *Δgal83* as well (Figures 2B,C) indicated cross-talk among these transcriptional regulators. To understand coordinated regulation of genes of the MUT pathway by these regulators, we carried out RNA seq analysis of *GS115 vs Δmxr1* and *GS115 vs Δtrm1* as well and compared their transcriptomes with those of *GS115 vs Δgcn5* and *GS115 vs Δgal83* (Supplementary File 1). The gene expression profiles of *Δgcn5, Δgal83*, *Δmxr1,* and *Δtrm1* relative to that of *GS115* is presented as Venn diagrams as well as heat maps in Figures 3, 4. Deletion of Gcn5, Gal83, Mxr1p, and Trm1p results in predominantly downregulation (Figure 3A, sector ABCD) rather than upregulation (Figure 3B, sector ABCD) of genes in cells cultured in YNBM indicating that they facilitate transcriptional activation than transcriptional repression. In this study, we focused our attention on the role of Gcn5 and Gal83 in facilitating trans-activation by Mxr1p and Trm1p and therefore focused our attention on genes which are downregulated in their absence (Figures 3A,C–E, 4). At least 37 genes are downregulated in *Δmxr1, Δtrm1*, *Δgcn5* as well as *Δgal83* (Figure 3A, sector ABCD) and these are listed in Figure 3C. Genes which are activated by Mxr1p or Trm1p but require Gcn5 and Gal83 as well for trans-activation are listed in Figures 3D,E. Of these, trans-activation of 13 genes is coordinately regulated by Mxr1p, Gcn5, and Snf1 but not Trm1p (marked by asterisk, Figure 3D). Similarly, trans-activation of 12 genes requires the participation of Gcn5, Snf1 and Trm1p but not Mxr1p (marked by asterisk, Figure 3E). Genes whose trans-activation requires Gcn5 as well as Gal83 are listed in Figure 4. Details of genes in each sector of the Venn diagram (Figure 3A) are provided in Supplementary File 2. To demonstrate the robustness of RNA seq data reported in this study, we have validated the expression of select genes downregulated in *Δgcn5* cultured in YNBM by qPCR and these data are provided in Supplementary File 3.

This study demonstrates for the first time that Gcn5, a HAT, and Snf1, a protein kinase, are key regulators of *K. phaffii* carbon metabolism in general and methanol metabolism in particular. In conjunction with two zinc finger transcription factors, Gcn5 and Snf1 regulate the expression of a plethora of genes during methanol metabolism. Comparative transcriptome analysis indicates extensive cross-talk among Mxr1p, Trm1p, Gcn5, and Snf1. Future investigations aimed at the study of the promoter occupancy by transcription factors and recruitment of chromatin modifiers in a promoter-specific manner is essential for understanding transcriptional regulation of genes of MUT pathway of *K. phaffii*. The identification of genes of MUT pathway that are coordinately regulated by multiple transcriptional regulators and chromatin modifiers should be followed up by the investigation of regulatory relationships among them. Recruitment of co-activators by transcription factors in a target-gene-specific manner is a key step in eukaryotic gene regulation. The ability of transcription factors such as Mxr1p and Trm1p to recruit chromatin modifiers such as Gcn5 and Snf1 to specific target gene promoters should be investigated by chromatin immunoprecipitation (ChIP) and/or ChIP sequencing. Gcn5-specific chromatin modifications occurring at the promoters of genes such as *AOX* can be studied in *GS115*, *Δmxr1,* and *Δtrm1* using anti-acetyl lysine histone antibodies against histone residues that are acetylated by Gcn5. In another approach, Snf1-mediated phosphorylation of Mxr1p and/or Trm1p can be studied by immunoprecipitating them from cell lysates of *GS115* and *Δgal83* followed by mass spectrometry and phosphopeptide mapping.

It is well known that trans-activation domains (TADs) of transcription factors play a critical role in the promoter-specific recruitment of co-activators. We have recently identified a 9 amino acid TAD (QELESSLNA) between amino acids 365 and 373 of Mxr1p which is required for the activation of genes essential for ethanol metabolism (Gupta et al., 2021). However, its role in the transcriptional regulation of Mxr1p target genes involved in methanol metabolism is not known. Preliminary investigations indicate that this TAD is required for the methanol-inducible trans-activation of *AOXI* by Mxr1p (P.N.R. unpublished data). It will be interesting to examine the role of this TAD in the recruitment of HATs such as Gcn5 to *AOX1* promoter. Further, Mxr1p harbors several putative TADs between 401 and 1,155 amino acids and several Mxr1p target genes which are activated by a truncated form of Mxr1p containing 400N-terminal amino acids (Mxr1-N400) or the full-length protein have been identified recently (Gupta et al., 2021). Among these, *FET4-1* and *YSF3* encoding a putative iron transporter and putative splicing factor, respectively, (Figure 3) are activated only by the full-length Mxr1p but not Mxr1-N400 indicating a key role for the putative TAD(s) located between amino acids 401 and 1,155 (Gupta et al., 2021). Interestingly, *FET4-1* and *YSF3* are among those genes that are co-regulated by Mxr1p, Gcn5 as well as Snf1 (Figure 3D). Characterization of these TADs and analysis of their role in the recruitment of Gcn5 to the promoters of *FET4-1* and *YSF3* are topics of future investigations.

In *S. cerevisiae*, Gal83 not only facilitates nuclear localization of Snf1 complex but also mediates the interaction of the Snf1 kinase complex with specific transcription factors such as Sip4, a transcription activator of gluconeogenic genes. Gal83 also facilitates rapid Snf1-dependent phosphorylation and activation of Sip4 (Vincent and Carlson, 1999). The results of this study indicate that Gal83 may have a crucial role in the interaction of Snf1 kinase complex with Mxr1p and Trm1p as well. Further, a direct role for Snf1 kinase in the phosphorylation of Mxr1p TADs cannot be ruled out at this stage. The fact that interactions among chromatin modifiers and other transcriptional regulators of MUT pathway, such as Mit1p, Mig1p, Mig2p, Nrg1p, and Rop1p, have not yet been investigated indicates that we have barely touched the tip of the iceberg.

## Data availability statement

The datasets presented in this study can be found in online repositories. The names of the repository/repositories and accession number(s) can be found in the article/Supplementary material.

## Author contributions

AG and PR conceived the project, designed the experiments, analyzed the results, and wrote the manuscript. AG carried out all the experiments. PR obtained funding. All authors contributed to the article and approved the submitted version.

## Funding

This work was supported by the J. C. Bose Fellowship grant SB/S2/JCB-025/2015 awarded by the Science and Engineering Research Board, New Delhi, India, and the research grant BT/PR30986/BRB/10/1751/2018 awarded by the Department of Biotechnology, New Delhi, India (to PR). Funding from the Department of Science and Technology Fund for Improvement of S&T Infrastructure in Higher Educational Institutions (DST-FIST), the University Grants Commission, and the Department of Biotechnology-Indian Institute of Science partnership program is acknowledged.

## Conflict of interest

The authors declare that the research was conducted in the absence of any commercial or financial relationships that could be construed as a potential conflict of interest.

## Publisher’s note

All claims expressed in this article are solely those of the authors and do not necessarily represent those of their affiliated organizations, or those of the publisher, the editors and the reviewers. Any product that may be evaluated in this article, or claim that may be made by its manufacturer, is not guaranteed or endorsed by the publisher.
