# Supplementary material for: Coordinate regulation of methanol utilization pathway genes of Komagataella phaffii by transcription factors and chromatin modifiers
Source: Front Microbiol. 2022 Sep 6;13:991192. doi: 10.3389/fmicb.2022.991192 (PMC9485576; doi:10.3389/fmicb.2022.991192)
Supplement: Supplementary file 1 [file Data_Sheet_1.PDF]

Supporting information 2

Details of genes listed in Fig. 3A

89 genes downregulated in only ∆mxr1 (sector A)

Id	Protein_name	Gene names (primary)	GI	UniRef100_Hit
gene2773	-	NA	NA	-
gene5407	Cell agglutination protein mam3	NA	NA	A0A1B2JED7
gene295	Conserved predicted protein	PP7435_Chr1-1862	NA	A0A1G4KP50
gene937	Protein localized to COPII vesicles, proposed to be involved in ER to Golgi transport	YIP3-2	YIP3-2	A0A1G4KPD2
gene1618	Papain-like cysteine prorease	PP7435_Chr1-1550	NA	A0A1G4KPN7
gene2806	ParB/Sulfiredoxin	SRX1	SRX1	A0A1G4KQ05
gene2772	Conserved predicted protein	PP7435_Chr2-1040	NA	A0A1G4KQ10
gene2916	Conserved predicted protein	PP7435_Chr2-1173	NA	A0A1G4KQ27
gene3496	Metallo-hydrolase / oxidoreductase	GLO2	GLO2	A0A1G4KQA9
gene4149	Polycystic kidney disease protein 1-like 3	PP7435_Chr3-1038	NA	A0A1G4KQL0
gene471	Haloacid dehalogenase-like hydrolase domain-containing protein 3	NA	NA	C4QW87
gene897	Protein tyrosine phosphatase superfamily	NA	NA	C4QXD7
gene1616	Cell surface glycoprotein 1	NA	NA	C4QZC5
gene2740	Potential RNAseP/MRP complex component	NA	NA	C4R085
gene1887	Core histone H2A/H2B/H3/H4	NA	NA	C4R2J6
gene1886	Core histone H2A/H2B/H3/H4	NA	NA	C4R2J7
gene4354	Genomic scaffold, Kuraishia_capsulata_scaffold_4	NA	NA	C4R3A3
gene3954	Mitochondrial peculiar membrane protein 1	NA	NA	C4R4B9
gene3532	Protein kinase-like (PK-like)	NA	NA	C4R5G6
gene5264	Papain-like cysteine prorease	NA	NA	C4R701
gene1937	FAD/NAD(P)-binding domain	NA	NA	C5NSJ4
gene0	Conserved predicted protein	PP7435_Chr1-0001	NA	F2QL95
gene6	High-affinity glucose transporter HGT1	GTH1	GTH1	F2QLA1
gene9	C6 transcription factor	PP7435_Chr1-0010	NA	F2QLA4

gene10	NAD(P)-binding Rossmann-fold domains	PP7435_Chr1-0011	NA	F2QLA5
gene998	E3 ubiquitin-protein ligase ARI5	HEL1	HEL1	F2QLI9
gene999	Thioredoxin-like	AHP1	AHP1	F2QLJ0
gene1021	Polyamine transporter that recognizes spermine, putrescine, and spermidine	TPO1	TPO1	F2QLL2
gene188	Essential protein involved in mtDNA inheritance	DML1	DML1	F2QM55
gene430	Glutamate/Leucine/Phenylalanine/Valine dehydrogenase	GDH3	GDH3	F2QMB9
gene727	Conserved predicted protein	PP7435_Chr1-0707	NA	F2QMP5
gene983	Peptidase inhibitor 15	PRY2	PRY2	F2QNB4
gene218	Alanyl-tRNA synthetase (Alanine-tRNA ligase) (AlaRS)	PP7435_Chr1-0207	NA	F2QND8
gene219	Genomic scaffold, Kuraishia_capsulata_scaffold_2	PP7435_Chr1-0208	NA	F2QND9
gene221	YVTN repeat-like/Quinoprotein amine dehydrogenase	CIA1	CIA1	F2QNE1
gene239	Beta-1,3-glucanosyltransferase, required forcell wall assembly	GAS1-2	GAS1-2	F2QNF9
gene294	Conserved predicted protein	PP7435_Chr1-0284	NA	F2QNL2
gene304	Pma1 plasma membrane H(+)-ATPase	PMA1	PMA1	F2QNM0
gene572	DNA Topoisomerase III, conserved protein that functions in a complex with Sgs1p and Rmi1p	TOP3	TOP3	F2QNW2
gene693	NADPH-dependent medium chain alcoholdehydrogenase with broad substrate specificity	ADH7	ADH7	F2QP78
gene1145	Acetyl-CoAC-acetyltransferase (Acetoacetyl-CoA thiolase), cytosolicenzyme	ERG10	ERG10	F2QP92
gene1286	Plasma membrane multidrug transporter of the major facilitator superfamily	FLR1	FLR1	F2QPM2
gene1307	Protein kinase-like (PK-like)	KIN3	KIN3	F2QPP2
gene1641	NAD(P)-binding Rossmann-fold domains	PP7435_Chr1-1573	NA	F2QQK5
gene1911	Thioredoxin-like	TSA1	TSA1	F2QR99
gene1929	Protein of unknown function (DUF1689)	PP7435_Chr2-0249	NA	F2QRB7
gene1954	Bud neck-localized, SH3domain-containing protein required for cytokinesis	HOF1	HOF1	F2QRD9
gene2102	Ammonium transporter MEP3	MEP1	MEP1	F2QRS3
gene2295	UDP-galactose transporter homolog 1	HUT1	HUT1	F2QS98
gene2377	DNA-directed DNA polymerase epsilon, subunit C	DPB3	DPB3	F2QSH2
gene2464	NAD(P)-binding Rossmann-fold domains	IRC24	IRC24	F2QSQ6
gene2467	C-jun-amino-terminal kinase-interacting protein 3	PP7435_Chr2-0752	NA	F2QSQ9
gene2521	GST C-terminal domain-like	URE2-2	URE2-2	F2QSV9
gene2610	RNA-binding, RBD	SGN1-1	SGN1-1	F2QT39
gene2679	Mitochondrial integral inner membrane protein required for membrane insertion of C-terminus of Cox2p	COX18	COX18	F2QTA3

gene2711	Uncharacterized membrane protein YLR326W	PP7435_Chr2-0980	NA	F2QTD3
gene2756	P-loop containing nucleosidetriphosphatehydrolases	SNQ2	SNQ2	F2QTH6
gene2834	Desmoglein-3	PP7435_Chr2-1097	NA	F2QTP6
gene2917	YVTN repeat-like/Quinoprotein amine dehydrogenase	PP7435_Chr2-1175	NA	F2QTX3
gene2977	Ankyrin repeat and KH domain-containing protein 1	PP7435_Chr2-1229	NA	F2QU26
gene3018	Hsp70 nucleotide exchange factor FES1	FES1	FES1	F2QU62
gene3228	D-lactate dehydrogenase, oxidizes D-lactateto pyruvate, transcription is heme-dependent	DLD1	DLD1	F2QUP2
gene3286	Vacuolar membrane zinc transporter	ZRT3	ZRT3	F2QUU8
gene3332	Origin recognition complex subunit 6 (ORC6)	ORC6	ORC6	F2QUY8
gene3348	Component of the mitotic spindle that binds to interpolar microtubules	STU1	STU1	F2QV03
gene3711	Molybdenum cofactor sulfurase protein (HxB)	PP7435_Chr3-0614	NA	F2QVZ3
gene3779	Protein with a role in regulation of Ty1transposition	RTT106	RTT106	F2QW58
gene3858	NAD(P)-binding Rossmann-fold domains	PP7435_Chr3-0762	NA	F2QWD8
gene3861	NAD(P)-binding Rossmann-fold domains	PP7435_Chr3-0765	NA	F2QWE1
gene3935	Widely conserved NADPH oxidoreductase containing flavin mononucleotide (FMN)	OYE3-1	OYE3-1	F2QWL2
gene3946	GPI-anchored wall transfer protein	GWT1	GWT1	F2QWM0
gene3953	Conserved ubiquitin-like modifier	ATG12	ATG12	F2QWM7
gene4039	Similar to uniprot P17260 Saccharomyces cerevisiae YNL322c KRE1 cell wall protein	PP7435_Chr3-0933	NA	F2QWV3
gene4058	Coenzyme Q (Ubiquinone) binding protein	COQ10	COQ10	F2QWW9
gene4164	Dicarboxylic amino acid permease	DIP5-2	DIP5-2	F2QX65
gene4313	Thiamine pyrophosphokinase, phosphorylates thiamine to produce the coenzyme thiaminepyrophosphate	THI80	THI80	F2QXK4
gene4541	Glycerol uptake facilitator protein	PP7435_Chr4-0171	NA	F2QY68
gene4542	Glycerol kinase, converts glycerol to glycerol-3-phosphate	GUT1	GUT1	F2QY70
gene4653	E3 ubiquitin-protein ligase ptr1	PP7435_Chr4-0279	NA	F2QYH6
gene4693	Signal recognition particle alu RNA binding heterodimer| srp9/1	PP7435_Chr4-0320	NA	F2QYL2
gene4788	Related to SPO75-Meiosis-specific protein required for spore wall formation during sporulation	SPO75	SPO75	F2QYV3
gene4850	Non-classical export protein 2	PP7435_Chr4-0473	NA	F2QZ11
gene4882	Member of the Sir2 family ofNAD(+)-dependent protein deacetylases	HST4	HST4	F2QZ40
gene4900	Conserved predicted protein	PP7435_Chr4-0519	NA	F2QZ55
gene4925	Plasma membrane transporter for both urea and polyamines, expression is highly sensitive to nitrogen	DUR3-3	DUR3-3	F2QZ78
gene4933	NAD(P)-linked oxidoreductase	PP7435_Chr4-0551	NA	F2QZ85

gene4994	Zonadhesin	PP7435_Chr4-0605	NA	F2QZD7
gene5165	Ferric reductase, reduces siderophore-bound iron prior to uptake by transporters	FRE3	FRE3	F2QZU3
gene5235	Isoamyl acetate-hydrolyzing esterase 1	IAH1	IAH1	F2R010

19 genes downregulated in Δmxr1 & Δgcn5 (sector AC)

Id	Protein_name	Gene names (primary)	GI	UniRef100_Hit
gene4779	AN1-type zinc finger protein 1	CUZ1	CUZ1	A0A1G4KQS6
gene1424	Conserved predicted protein	NA	NA	C4QYU2
gene2856	Heme Oxygenase| Chain A	NA	NA	C4QZX3
gene2791	NAD(P)-binding Rossmann-fold domains	NA	NA	C4R037
gene4825	Cell wall protein TIR3	NA	NA	C4R859
gene4798	NAD(P)-linked oxidoreductase	NA	NA	C4R885
gene1057	2-enoyl-CoA Hydratase| Chain A| domain 1	ECI1	ECI1	F2QLP4
gene613	NAD(P)-binding Rossmann-fold domains	SOR1	SOR1	F2QP04
gene1156	Likely SIR2 family histonedeacetylase similar to S. cerevisiae HST2 (YPL015C) involved in telomeric chromatin silencing	HST2	HST2	F2QPA3
gene1340	Conserved predicted protein	PP7435_Chr1-1283	NA	F2QPS4
gene1403	Acyl-coenzyme A oxidase	POX1	POX1	F2QPY1
gene1799	Cytochrome c peroxidase	CCP1-1	CCP1-1	F2QR02
gene1962	3-ketoacyl-CoA thiolase with broad chain length specificity	POT1	POT1	F2QRE5
gene2388	Nitrosoguanidine resistance protein SNG1	SNG1	SNG1	F2QSI1
gene3901	Alternative oxidase	AOX100	AOX100	F2QWI0
gene3936	Widely conserved NADPH oxidoreductase containing flavin mononucleotide (FMN)	OYE3-1	OYE3-1	F2QWL2
gene3937	Widely conserved NADPH oxidoreductase containing flavin mononucleotide (FMN)	OYE3-1	OYE3-1	F2QWL2
gene3938	Widely conserved NADPH oxidoreductase containing flavin mononucleotide (FMN)	OYE3-2	OYE3-2	F2QWL3
gene5058	Type I PLP-dependent aspartate aminotransferase-like (Major domain)	MET17	MET17	F2QZJ5

11 genes downregulated in Δmxr1, Δgcn5 & Δtrm1 (sector ACD)

Id	Protein_name	Gene names (primary)	GI	UniRef100_Hit
gene5198	Ribose-5-phosphate isomerase	RKI1-2	RKI1-2	A0A1G4KQZ7

gene1074	Altered inheritance rate of mitochondria protein 38	RCF2	RCF2	F2QLQ9
gene162	Plasma membrane pyridoxine (VitaminB6)transporter	TPN1	TPN1	F2QM34
gene764	Transmembrane protein involved in export of ammonia	ADY2-2	ADY2-2	F2QMS4
gene655	Fructose-bisphosphate aldolase variant 1, class II	FBA1-2	FBA1-2	F2QP45
gene1155	Farnesyl pyrophosphate synthase	ERG20	ERG20	F2QPA2
gene1425	Actin-like ATPase domain	HXK1	HXK1	F2QQ02
gene1928	Homeobox transcription factor	PP7435_Chr2-0248	NA	F2QRB6
gene3172	GST C-terminal domain-like	PP7435_Chr3-0094	NA	F2QUJ0
gene3400	alpha/beta-Hydrolases	FGH1	FGH1	F2QV54
gene3868	Ribulose-phosphate 3-epimerase	RPE1-2	RPE1-2	F2QWE8

30 genes downregulated in ∆mxr1 & ∆trm1 (sector AD)

Id	Protein_name	Gene names (primary)	GI	UniRef100_Hit
gene4817	Tubulin beta chain	TUB2	TUB2	A0A1G4KQS3
gene241	Cell wall protein with similarity to glucanases	NA	NA	C4QVL7
gene2563	(Trans)glycosidases	NA	NA	C4R0Q7
gene2206	Protein with internal repeats 1	NA	NA	E0A3M5
gene1096	Protein required for maturation of the 25S and 5.8S ribosomal RNAs	PP7435_Chr1-1042	NA	F2QLE0
gene119	Conserved predicted protein	PP7435_Chr1-0116	NA	F2QLZ5
gene238	Beta-1,3-glucanosyltransferase, required for cell wall assembly	GAS1-1	GAS1-1	F2QNF8
gene656	PH domain-like protein	PP7435_Chr1-0640	NA	F2QP46
gene1243	Ammonium transporter MEP2	MEP2	MEP2	F2QPI2
gene1281	Glucan 1,3-beta-glucosidase BGL2	BGL2	BGL2	F2QPL8
gene1595	G1/S-specific cyclin CLN1	CLN2	CLN2	F2QQG3
gene1728	Plasma membrane multidrug transporter of the major facilitator superfamily	AQR1	AQR1	F2QQT9
gene1788	Smi1	PP7435_Chr2-0123	NA	F2QQZ2
gene1789	Genomic scaffold, Kuraishia_capsulata_scaffold_1	PP7435_Chr2-0124	NA	F2QQZ3
gene1819	Methylenetetrahydrofolate reductase	MET13-1	MET13-1	F2QR20
gene2962	Conserved predicted protein	PP7435_Chr2-1215	NA	F2QU12
gene3008	P-loop containing nucleosidetriphosphatehydrolases	STE6-3	STE6-3	F2QU54

gene3395	Mannose-1-phosphateguanyl transferase (ATP-man nose-1-phosphateguanylyltransferase) (GDP-mannosepyrophosphorylase)(CASRB1)	PSA1-1	PSA1-1	F2QV49
gene3409	Phospholipidmethyltransferase (Methylene-fatt y-acyl-phospholipidsynthase)	OPI3	OPI3	F2QV61
gene3521	Spore-specific water channel that mediates the transport of water across cell membranes	AQY1	AQY1	F2QVG4
gene3725	Peroxisomal nudix pyrophosphatase with specificity for coenzyme A and CoA derivatives	PCD1	PCD1	F2QW07
gene3812	Conserved predicted protein	PP7435_Chr3-0714	NA	F2QW92
gene3863	alpha/beta-Hydrolases	PP7435_Chr3-0767	NA	F2QWE3
gene4137	WD repeat and HMG-box DNA-binding protein 1	CTF4	CTF4	F2QX44
gene4154	Major of three pyruvate decarboxylase isozymes	PDC1	PDC1	F2QX59
gene4165	Malic enzyme, NAD binding domain	MAE1	MAE1	F2QX66
gene4365	Conserved predicted protein	PP7435_Chr4-0069	NA	F2QXW9
gene4789	Concanavalin A-like lectins/glucanases	CRH1	CRH1	F2QYV4
gene5328	Conserved predicted protein	PP7435_Chr4-0927	NA	F2R0A0
gene5381	Conserved predicted protein	PP7435_Chr4-0976	NA	F2R0E8

15 genes downregulated in ∆mxr1&∆gal83 (sector AB)

Id	Protein_name	Gene names (primary)	GI	UniRef100_Hit
gene962	Pirin-like protein	NA	NA	C4QXK1
gene1406	Separase, a caspase-like cysteine proteas	NA	NA	C4QYS2
gene3006	Family 17 glucosidase SCW11 (Soluble cell wall protein 11)	NA	NA	C4QZH9
gene441	Ribonuclease H2 subunit B	RNH202	RNH202	F2QMC9
gene1803	Peroxisomal catalase	CTA1	CTA1	F2QR06
gene2188	Homeodomain-like	YHP1	YHP1	F2QRZ5
gene2635	Arginine/alanine aminopeptidase	PP7435_Chr2-0908	NA	F2QT62
gene2673	YVTN repeat-like/Quinoprotein amine dehydrogenase	DSE1	DSE1	F2QT98
gene2853	Meiotic sister chromatid recombination protein 1	PP7435_Chr2-1115	NA	F2QTR4
gene2877	Subtilisin-like serine protease pr1c	SBT100	SBT100	F2QTT6
gene3062	Alpha, alpha-trehalose-phosphatesynthase (UDP-forming)	TPS1	TPS1	F2QUA0
gene3241	Muscle M-line assembly protein unc-89	PP7435_Chr3-0161	NA	F2QUQ5
gene3247	(Trans)glycosidases	CTS1	CTS1	F2QUR1

gene4334	Zonadhesin	PP7435_Chr3-1213	NA	F2QXM5
gene5418	Proton-coupled oligopeptide transporter of the plasma membrane	OPT1-1	OPT1-1	F2R0I2

13 genes downregulated in Δmxr1, Δgal83 & Δgcn5 (sector ABC)

Id	Protein_name	Gene names (primary)	GI	UniRef100_Hit
gene3646	-	NA	NA	-
gene4340	High-affinity glucose transporter	NA	NA	A0A1B2J7E9
gene586	Membrane protein	PP7435_Chr1-0569	NA	F2QNX7
gene1378	Sterol uptake protein 2	SUT2	SUT2	F2QPV7
gene2187	Long chain fatty acyl-CoA synthetase with a preference for C12:0-C16:0 fatty acids	FAA1	FAA1	F2QRZ4
gene3176	Translation machinery-associated protein 17	TMA17	TMA17	F2QUJ4
gene3201	P-loop containing nucleosidetriphosphatehydrolases	PEX1	PEX1	F2QUL6
gene3647	Thiamine thiazole synthase	THI4	THI4	F2QVT2
gene4091	Genomic scaffold, Kuraishia_capsulata_scaffold_1	TFB6	TFB6	F2QX02
gene4296	Peptide methionine sulfoxidereductase, reverses the oxidation of methionine residues	MXR1-1	MXR1-1	F2QXI9
gene5050	Membrane protein	PP7435_Chr4-0658	NA	F2QZI8
gene5206	RNA polymerase II transcription factor B subunit 3 (RNA polymerase II transcription factor B p38 subunit) (RNA polymerase II transcription factor B 38kDa subunit)	TFB3	TFB3	F2QZY0
gene1871	Peroxisomal targeting signal 1 receptor	NA	NA	P33292

37 genes downregulated in Δmxr1, Δgal83, Δgcn5 & Δtrm1 (sector ABCD)

Id	Protein_name	Gene names (primary)	GI	UniRef100_Hit
gene3439	-	NA	NA	-
gene1412	Peroxisomal membrane associated protein 20	PMP20	PMP20	A0A1G4KPJ6
gene2970	Dur3 urea transporter	NA	NA	C4QZL5
gene2492	Calcium-binding mitochondrial carrier protein SCaMC-1	NA	NA	C4R0X5
gene3942	Internalin-I	NA	NA	C4R4D1
gene8	FAD/NAD(P)-binding domain	FMO1-1	FMO1-1	F2QLA3
gene1122	alpha/beta-Hydrolases	PP7435_Chr1-1067	NA	F2QLG5

gene717	Transmembrane protein involved in export of ammonia	ADY2-4	ADY2-4	F2QMN6
gene868	G1 cyclin involved in cell cycleprogression	CLN3-1	CLN3-1	F2QN12
gene906	Polyamine transporter 3	TPO3	TPO3	F2QN43
gene255	Flavin adenine dinucleotide (FAD)synthetase	FAD1	FAD1	F2QNH4
gene585	Splicing factor 3B subunit 5	YSF3	YSF3	F2QNX6
gene618	Plasma membrane permease proposed to be involved in carboxylic acid uptake	THI73	THI73	F2QP09
gene1394	Conserved predicted protein	PEX11C	PEX11C	F2QPX2
gene1662	Low-affinity Fe(II) transporter of the plasma membrane	FET4-1	FET4-1	F2QQM7
gene1782	Inositol 1-phosphate synthase	INO1	INO1	F2QQY8
gene1861	Phosphoglycerate mutase-like protein	SHB17	SHB17	F2QR54
gene1898	Conserved predicted protein	PP7435_Chr2-0218	NA	F2QR87
gene2049	Transaldolase	TAL1-2	TAL1-2	F2QRM4
gene2050	Transaldolase, enzyme in the non-oxidative pentose phosphate pathway	TAL1-2	TAL1-2	F2QRM4
gene2233	Glutathione transferase	PP7435_Chr2-0527	NA	F2QS36
gene2284	Acyl-coenzyme A thioesterase 9	PP7435_Chr2-0578	NA	F2QS87
gene2350	Genomic scaffold, Kuraishia_capsulata_scaffold_4	PP7435_Chr2-0638	NA	F2QSE7
gene2507	Peroxisomal membrane protein 11A	PEX11	PEX11	F2QSU6
gene3430	Thiamin biosynthetic bifunctional enzyme	THI6	THI6	F2QV82
gene3431	Multifunctional protein with both hydroxymethylpyrimidine kinase and thiaminase activities	THI20	THI20	F2QV83
gene3440	Dihydroxyacetone synthase (DHAS) (TKL2) (Formaldehyde transketolase) (Glycerone synthase)	DAS2	DAS2	F2QV90
gene3442	Dihydroxyacetone synthase variant 1	DAS1	DAS1	F2QV92
gene4110	FAD/NAD(P)-binding domain	OSM1	OSM1	F2QX19
gene4476	Glyoxalase/Bleomycin resistance protein/Dihydr oxybiphenyl dioxygenase	GLO1	GLO1	F2QY07
gene4497	Alcohol oxidase 1	AOX1	AOX1	F2QY27
gene4839	Low-affinity zinc transporter of the plasma membrane	ZRT2	ZRT2	F2QYZ9
gene5129	Cytochrome b2 (L-lactatecytochrome-c oxidoreductase)	CYB2	CYB2	F2QZQ8
gene5263	Alcohol oxidase 1	AOX2	AOX2	F2R038
gene5305	Protein that associates with ribosomes	TMA108	TMA108	F2R077
gene3432	Dihydroxyacetone kinase, required for detoxification of dihydroxyacetone (DHA)	NA	NA	O74192
gene1192	Peroxisomal biogenesis factor 8	NA	NA	Q01962

21 genes downregulated in Δmxr1, Δgal83 & Δtrm1 (sector ABD)

Id	Protein_name	Gene names (primary)	GI	UniRef100_Hit
gene4134	Protein lin-49	NA	NA	-
gene203	Holo	PPT2	PPT2	A0A1G4KP41
gene484	Actin patches distal protein 1	PP7435_Chr1-2104	NA	A0A1G4KP73
gene1293	FMN dependent fluorescent proteins	NA	NA	C4QYG5
gene1509	Flap endonuclease 1	NA	NA	C4QZ20
gene1948	Cell surface glycoprotein 1	NA	NA	C4R2D7
gene1726	Protein of the SUN family (Sim1p,Uth1p,Nca3p, Sun4p) that may participate in DNA replication	NA	NA	C4R2Z5
gene4004	Conserved predicted protein	NA	NA	C4R474
gene604	Protein tyrosine phosphatase involved in cell cycle control	MIH1	MIH1	F2QNZ5
gene1655	Contains GLEYA adhesin domain	FLO100	FLO100	F2QQL9
gene1989	Tetratricopeptide repeat protein 15	PP7435_Chr2-0306	NA	F2QRH2
gene2044	P-loop containing nucleotidetriphosphatehydrolases	YFH7	YFH7	F2QRL9
gene2285	Integral plasma membrane protein required for axial budding in haploid cells	AXL2	AXL2	F2QS88
gene2294	Conserved predicted protein	PP7435_Chr2-0588	NA	F2QS97
gene2429	NAD(P)-linked oxidoreductase	PP7435_Chr2-0714	NA	F2QSM2
gene2760	Coiled-coil domain-containing protein 67	SLK19	SLK19	F2QTI0
gene2965	Pho85 cyclin of the Pcl1, 2-like subfamily, involved in entry into the mitotic cell cycle and regulation	PCL1	PCL1	F2QU15
gene2976	Structural maintenance of chromosome proteins	SMC1	SMC1	F2QU25
gene4283	Venom allergen 5.01	EPX1	EPX1	F2QXH5
gene4699	Glycoside hydrolase family 45 protein	RCE3	RCE3	F2QYL8
gene5227	Absent in melanoma 1 protein	PP7435_Chr4-0827	NA	F2R002

62 genes downregulated only in Δgal83 (sector B)

Id	Protein_name	Gene names (primary)	GI	UniRef100_Hit
gene3168	Predicted protein	NA	NA	-
gene754	-	NA	NA	-
gene755	-	NA	NA	-

gene3325	Uncharacterized WD repeat-containing protein alr3466	NA	NA	-
gene4989	-	NA	NA	-
gene453	Trimethyl lysine dioxygenase(Epsilon-trimethyl lysine 2-oxoglutaratedioxygenase)(TML-alpha-ketoglutarate dioxygenase) (TMLhydroxylase)(TML dioxygenase) (TMLD)	TML1	TML1	A0A1G4KP69
gene1723	NAD(P)-binding Rossmann-fold domains	AYR1	AYR1	A0A1G4KPN4
gene2509	Thiol-specific peroxiredoxin, reduces hydroperoxides to protect against oxidative damage	PRX5	PRX5	A0A1G4KPY0
gene4366	Proteolipid membrane potential modulator	PP7435_Chr4-1147	NA	A0A1G4KQP4
gene367	Zinc-regulated transcription factor, binds to zinc-responsive promoter elements	NA	NA	C4QVZ1
gene1360	One of three possible beta-subunits of the Snf1 kinase complex	NA	NA	C4QYM8
gene1594	Transcriptional coactivator HFI1/ADA1	NA	NA	C4QZA4
gene2422	Isocitrate dehydrogenase	NA	NA	C4R142
gene4086	Serine/threonine-protein kinase	NA	NA	C4R3Z4
gene3734	Biotin:apoprotein ligase, covalentlym odifies proteins with the addition of biotin	NA	NA	C4R4Y1
gene78	Glycerol-3-phosphate/dihydroxyacetonephosphate dual substrate-specific sn-1 acyltransferase	GPT2	GPT2	F2QLV7
gene440	Laminin subunit gamma-1	PP7435_Chr1-0419	NA	F2QMC8
gene443	C2 domain (Calcium/lipid-binding domain, CaLB)	TCB3	TCB3	F2QMD1
gene480	Nuclear thiol peroxidase, functions as an alkyl-hydroperoxide reductase during post-diauxic growth	DOT5	DOT5	F2QMH0
gene482	Glutamate racemase	PP7435_Chr1-0467	NA	F2QMH2
gene765	Transmembrane protein involved in export of ammonia	ADY2-3	ADY2-3	F2QMS5
gene926	Protein involved in iron metabolism in mitochondria	NFU1-1	NFU1-1	F2QN61
gene973	Mediator of RNA polymerase II transcription subunit 15	GAL11	GAL11	F2QNA4
gene594	Interferon-related developmental regulator1	PP7435_Chr1-0578	NA	F2QNY5
gene690	MFS general substrate transporter like domains	DAL5-2	DAL5-2	F2QP75
gene1151	Ubiquitin-like modifier-activating enzyme ATG7	ATG7	ATG7	F2QP98
gene1208	Iron-regulated transcriptional activator AFT1	PpAFT1	PpAFT1	F2QPE8
gene1237	Domain-containing protein YLL032C	PP7435_Chr1-1175	NA	F2QPH6
gene1296	B-type cyclin involved in cell cycle progression	CLN3-2	CLN3-2	F2QPN1
gene1458	Conserved predicted protein	PP7435_Chr1-1393	NA	F2QQ31
gene1609	Phosphoenolpyruvate carboxykinase	PCK1	PCK1	F2QQH5
gene1796	Eukaryotic integral membrane protein(DUF1751)	PP7435_Chr2-0130	NA	F2QQZ9
gene2511	DNA polymerase alpha subunit B N-terminal	POL12	POL12	F2QSU9

gene2541	Alcohol dehydrogenase GroES-like domain	ADH2	ADH2	F2QSX6
gene2743	Secondary thiamine-phosphate synthase enzyme	PP7435_Chr2-1013	NA	F2QTG3
gene2942	Fructose-2,6-bisphosphatase, required for glucose metabolism	FBP26	FBP26	F2QTZ5
gene2954	G2/mitotic-specific cyclin CYB1	CLB2	CLB2	F2QU05
gene3135	Glycerol proton symporter of the plasma membrane, subject to glucose-induced inactivation	STL1-3	STL1-3	F2QUF7
gene3169	Conserved predicted protein	PP7435_Chr3-0091	NA	F2QUI7
gene3240	YVTN repeat-like/Quinoprotein amine dehydrogenase	PP7435_Chr3-0160	NA	F2QUQ4
gene3287	Glutamate decarboxylase	GAD1	GAD1	F2QUU9
gene3315	Subunit of the origin recognition complex, which directs DNA replication	ORC2	ORC2	F2QUX5
gene3356	Transcription factor, has homolog in Kluyveromyces lactis	SEF1	SEF1	F2QV09
gene3369	Potential protein lysine methyltransferase SET5	SET5	SET5	F2QV22
gene3459	Subunit g of the mitochondrial F1F0 ATP synthase	ATP20	ATP20	F2QVA7
gene3574	Divalent metal ion transporter involved in iron homeostasis	SMF2	SMF2	F2QVL5
gene3869	Carboxylic acid transporter protein	JEN1	JEN1	F2QWE9
gene3904	Genomic scaffold, Kuraishia_capsulata_scaffold_5	PP7435_Chr3-0808	NA	F2QWI3
gene4271	Similar to Altered inheritance of mitochondria protein 6 acc. no. C5DYR9	PP7435_Chr3-1148	NA	F2QXG3
gene4471	Self-glucosylating initiator of glycogen synthesis, also glucosylates n-dodecyl-beta-D-maltoside	GLG1	GLG1	F2QY02
gene4490	Thioesterase/thiol ester dehydrase-isomerase	MRX3	MRX3	F2QY20
gene4571	C3HC4-type RING-finger peroxisomal membrane peroxin	PEX12	PEX12	F2QY97
gene4715	Heat shock protein 9/12	HSP12	HSP12	F2QYN4
gene4808	Thiamine repressible genes regulatory protein thi1	CAT8-2	CAT8-2	F2QYX3
gene4898	Pig-x / pbn1	PBN1	PBN1	F2QZ53
gene5038	Potential peroxisomal adeninenucleotidetransporter protein	ANT1	ANT1	F2QZH7
gene5161	Chromosome segregation in meiosis protein 3	CSM3	CSM3	F2QZT9
gene5350	Histone chaperone ASF1	ASF1	ASF1	F2R0C0
gene5377	Aldehyde Dehydrogenase| Chain A| domain 1	ALD6-1	ALD6-1	F2R0E4
gene5412	MFS transporter, sugar porter (SP) family	MAL31	MAL31	F2R0H7
gene5425	Contains GLEYA adhesin domain	FLO300	FLO300	F2R0J1
gene4859	Peroxisomal ubiquitin conjugating enzyme	NA	NA	P49428

**16 genes downregulated in Δgal83 & Δgcn5 (sector BC)**				
Id	Protein_name	Gene names (primary)	GI	UniRef100_Hit
gene1099	Predicted protein	NA	NA	-
gene4527	Peroxisomal membrane peroxin that is a central component of the peroxisomal protein import machinery	NA	NA	A0A1B2JHV7
gene1441	Peroxisome biogenesis factor 10	PpPEX10	PpPEX10	A0A1G4KPJ1
gene4360	Predicted protein	MATa2	MATa2	A0A1G4KQN4
gene2431	FMN-binding split barrel	NA	NA	C4R133
gene4491	Myo-inositol transporter with strong similarity to the minor myo-inositol transporter I tr2p	NA	NA	C4R922
gene45	Biotin synthase, catalyzes the conversion of dethiobiotin to biotin	BIO2	BIO2	F2QLS8
gene405	Uncharacterized membrane protein YDL218W	PP7435_Chr1-0387	NA	F2QM97
gene738	Zinc finger, C3HC4 type (RING finger)	PP7435_Chr1-0718	NA	F2QMQ6
gene540	Genomic scaffold, Kuraishia_capsulata_scaffold_1	PP7435_Chr1-0522	NA	F2QNT1
gene2887	DNA polymerase delta catalytic subunit	POL3	POL3	F2QTU6
gene4358	Agglutinin-like protein 3	ALS3	ALS3	F2QXP8
gene4468	Conserved predicted protein	PP7435_Chr4-0101	NA	F2QXZ9
gene4503	lactate/malate dehydrogenase, NAD binding domain	MDH3	MDH3	F2QY33
gene4755	Conserved predicted protein	PP7435_Chr4-0381	NA	F2QYS1
gene4964	Translation machinery-associated protein 10	TMA10	TMA10	F2QZB2

**12 genes downregulated in Δgal83, Δgcn5 & Δtrm1 (sector BCD)**				
Id	Protein_name	Gene names (primary)	GI	UniRef100_Hit
gene3219	-	NA	NA	-
gene347	FAD/NAD(P)-binding domain	NA	NA	C4QVX2
gene1413	Vacuolar proteinase B (YscB), a serine protease of the subtilisin family	NA	NA	C4QYT0
gene2642	Homoserine O-acetyltransferase	NA	NA	C4R0I1
gene3404	Autophagy-related protein 30	NA	NA	C4R5T1
gene4742	Acyl-CoA-binding domain-containing protein 5	NA	NA	C4R8D7
gene313	Beta subunit of fatty acid synthetase, catalyzes the synthesis of long-chain saturated fatty acids	FAS1	FAS1	F2QNM8
gene1897	Integral peroxisomal membrane required for the translocation of peroxisomal matrix proteins	PEX13	PEX13	F2QR86
gene3324	NAD(P)-binding Rossmann-fold domains	FDH1	FDH1	F2QUY2

gene3438	Uncharacterized transcriptional regulatory protein YLL054C	PP7435_Chr3-0349	NA	F2QV89
gene4262	Mitochondrial NAD+ transporter, involved in the transport of NAD+ into the mitochondria (See alsoYE)	PMP47	PMP47	F2QXF4
gene3220	S-(hydroxymethyl) glutathionedehydrogenase/cl alcohol dehydrogenase	NA	NA	O74685

21 genes downregulated in Δgal83 & Δtrm1 (sector BD)

Id	Protein_name	Gene names (primary)	GI	UniRef100_Hit
gene1390	Histone H2A.Z-specific chaperone CHZ1	NA	NA	-
gene3468	Conserved predicted protein	PP7435_Chr3-1743	NA	A0A1G4KQA4
gene4231	Protein kinase-like (PK-like)	PP7435_Chr3-2810	NA	A0A1G4KQM4
gene869	JmjC domain-containing protein 4	PP7435_Chr1-0830	NA	F2QN13
gene616	ribonucleoside-diphosphate reductase, alpha subunit	RNR1	RNR1	F2QP07
gene624	Conserved predicted protein	PP7435_Chr1-0609	NA	F2QP16
gene1207	DNA replication licensing factor, MCM6 component	MCM6	MCM6	F2QPE7
gene1570	Vitamin H transporter 1	TNA1-2	TNA1-2	F2QQD8
gene1664	Topoisomerase 1-associated factor 1	TOF1	TOF1	F2QQM9
gene1975	Uricase	URO1	URO1	F2QRF8
gene1976	Mitochondrial N-glycosylase/DNAlyas	OGG1	OGG1	F2QRF9
gene2281	Required for respiratory growth protein 7	PP7435_Chr2-0575	NA	F2QS84
gene2498	Subunit of DNA primase,which is required for DNA synthesis and double-strand break repair	PRI2	PRI2	F2QST8
gene2900	DNA replication licensing factor mcm3	MCM3	MCM3	F2QTV9
gene3165	MFS transporter, sugar porter (SP) family	STL1-4	STL1-4	F2QUI4
gene3299	Uroporphyrinogen decarboxylase (URO-D)	HEM12	HEM12	F2QUW1
gene3641	P-loop containing nucleosidetriphosphatehydrolases	CTF18	CTF18	F2QVS7
gene4785	Immunoglobulin A1 protease	BNI4	BNI4	F2QYV0
gene5064	Eukaryotic and archaeal DNA primase small subunit	PRI1	PRI1	F2QZK0
gene5185	DNA replication licensing factor mcm2	MCM2	MCM2	F2QZW1
gene5213	Ribonucleoside-diphosphate reductase small chain 1	RNR2	RNR2	F2QZY7

57 genes downregulated in Δgcn5 (sector C)

Id	Protein_name	Gene names (primary)	GI	UniRef100_Hit
gene4562	Predicted protein	NA	NA	-
gene4578	NAD(P)-binding Rossmann-fold domains	NA	NA	A0A1B2JIU7
gene13	Conserved predicted protein	PP7435_Chr1-0014	NA	A0A1G4KP24
gene414	Conserved predicted protein	PP7435_Chr1-2038	NA	A0A1G4KP70
gene4498	Uncharacterized protein YDR514C and Good for full DBP5 activity protein 2	PP7435_Chr4-0131	NA	A0A1G4KQQ1
gene4965	Conserved predicted protein	PP7435_Chr4-0579	NA	A0A1G4KQV1
gene176	cAMP-independent regulatory protein pac2	NA	NA	C4QVF4
gene305	Permease, Suppressor of sulfoxydeethionine Resistance Vitamin H transporter (H+/biotinsymporter)	NA	NA	C4QVT0
gene750	Respiratory growth induced protein 1	NA	NA	C4QX11
gene1137	GTP cyclohydrolase URC1	NA	NA	C4QY14
gene1275	Homocitrate synthase isozyme, catalyzes the condensation of acetyl-CoA and alpha-ketoglutarate	NA	NA	C4QYE7
gene1361	Mitochondrial distribution and morphology protein 34	NA	NA	C4QYM9
gene2668	NAD(P)-binding Rossmann-fold domains	NA	NA	C4R0F6
gene2105	SPFH domain/Band 7 family protein	NA	NA	C4R1Z0
gene4890	Aldehyde Dehydrogenase| Chain A| domain 1	NA	NA	C4R7Z7
gene7	Isochorismatase family hydrolase	PP7435_Chr1-0008	NA	F2QLA2
gene1015	Nitric oxide oxidoreductase, flavo hemoglobin involved in nitric oxide detoxification	YHB1	YHB1	F2QLK6
gene1044	Genomic scaffold, Kuraishia_capsulata_scaffold_4	PP7435_Chr1-0991	NA	F2QLN2
gene419	Cyclin, interacts with Pho85p cyclin-dependent kinase (Cdk), induced by Gcn4p atlevel of transcript	PCL5	PCL5	F2QMB0
gene431	Nonspecific Lipid-transfer Protein| Chain A	POX18	POX18	F2QMC0
gene501	Lon protease homolog 2, peroxisomal	PIM1-2	PIM1-2	F2QMI9
gene733	Thiamin diphosphate-binding fold (THDP-binding)	PP7435_Chr1-0713	NA	F2QMQ1
gene769	Conserved predicted protein	PP7435_Chr1-0742	NA	F2QMS9
gene780	Mitochondrial peroxiredoxin (1-Cys Prx) with thioredoxin peroxidase activity	PRX1-2	PRX1-2	F2QMT8
gene949	P-loop containing nucleosidetriphosphatehydrolases	PEX6	PEX6	F2QN80
gene1215	C-5 sterol desaturase, catalyzes the introduction of a C-5(6) double bond into episterol	ERG3	ERG3	F2QPF5
gene1242	Gamma-butyrobetainedioxygenase (Gamma-butyrob etaine,2-oxoglutaratedioxygenase)(Gamma-butyrobetaine hydroxylase) (Gamma-BBH)	PP7435_Chr1-1181	NA	F2QPI1
gene1563	Conserved predicted protein	PP7435_Chr1-1496	NA	F2QQD1
gene1635	Alkaline dihydroceramidase, involved in sphingolipid metabolism	YPC1	YPC1	F2QQJ9

gene1649	MFS general substrate transporter like domains	MCH5-2	MCH5-2	F2QQL4
gene1674	Ubiquitin-binding component of the Rsp5pE3-ubiquitin ligase complex, functional homolog of Bul2p	BUL1	BUL1	F2QQN8
gene1895	WW domain containing protein interacting with Metacaspase (MCA1)	PP7435_Chr2-0215	NA	F2QR84
gene1972	Plasma membrane sulfite pump involved in sulfite metabolism	SSU1	SSU1	F2QRF5
gene2017	3-hydroxyanthranilate 3,4-dioxygenase	BNA1	BNA1	F2QRJ7
gene2191	Psp1 family protein	PP7435_Chr2-0488	NA	F2QRZ8
gene3103	Histone acetyltransferase, acetylates N-terminal lysines on histones H2B and H3	GCN5	GCN5	F2QUD1
gene3318	3-hydroxyisobutyryl-CoA hydrolase, member of a family of enoyl-CoA hydratase/isomerases	EHD3	EHD3	F2QUX8
gene3378	Subunit of a heterodimeric NC2 transcription regulator complex with Bur6p	NCB2	NCB2	F2QV32
gene3412	FAD/NAD(P)-binding domain	FMO1-4	FMO1-4	F2QV64
gene3511	BZIP transcription factor	MET4	MET4	F2QVF6
gene3988	Conserved predicted protein	PP7435_Chr3-0880	NA	F2QWQ4
gene3989	Tat pathway signal sequence domain protein	PP7435_Chr3-0881	NA	F2QWQ5
gene4021	Acid proteases	YPS1-4	YPS1-4	F2QWT5
gene4070	Classic Zinc Finger	PP7435_Chr3-0964	NA	F2QWY2
gene4217	ER localized, heme-binding peroxidase involved in the degradation of heme	HMX1	HMX1	F2QXB2
gene4312	C-4 methyl sterol oxidase, catalyzes the first of three steps required to remove two C-4 methyl groups	ERG25	ERG25	F2QXK3
gene4479	NAD(P)-binding Rossmann-fold domains	GAL10	GAL10	F2QY10
gene4822	Type I PLP-dependent aspartate aminotransferase-like (Major domain)	AAT2	AAT2	F2QYY5
gene4860	Peroxisomal membrane protein PEX26	PP7435_Chr4-0482	NA	F2QZ20
gene4891	Genomic scaffold, Kuraishia_capsulata_scaffold_3	PP7435_Chr4-0511	NA	F2QZ47
gene5037	YVTN repeat-like/Quinoprotein amine dehydrogenase	PP7435_Chr4-0646	NA	F2QZH6
gene5043	Similarto1-(5-phosphoribosyl)-5	HIS6	HIS6	F2QZI1
gene5044	Acid trehalase required for utilization of extracellular trehalose	ATH1	ATH1	F2QZI2
gene5262	Phospholipase B (Lysophospholipase) involved in phospholipid metabolism	PLB3	PLB3	F2R037
gene5269	Type I PLP-dependent aspartate aminotransferase-like (Major domain)	ARO9	ARO9	F2R043
gene5378	Septicolysin	PP7435_Chr4-0973	NA	F2R0E5
gene4324	Peroxisomal biogenesis factor 2	NA	NA	Q01964

14 genes downregulated in Δgcn5 & Δtrm1 (sector CD)

Id	Protein_name	Gene names (primary)	GI	UniRef100_Hit
gene676	Transcription factor TOS4	PP7435_Chr1-2247	NA	A0A1G4KP85
gene3749	Zinc-finger protein involved in transcriptional control of both nuclear and mitochondrial genes	NA	NA	C4R4W8
gene161	Glucose repression protein GAL83	UIP4	UIP4	F2QM33
gene797	Pyridoxine (Pyridoxamine) phosphateoxidase, has homologs in E. coli and Myxococcus xanthus	PDX3	PDX3	F2QMV2
gene1316	Malate synthase G	DAL7	DAL7	F2QPQ1
gene3229	F-box/LRR-repeat protein 15	PP7435_Chr3-0149	NA	F2QUP3
gene3313	Coproporphyrinogen III oxidase, anoxygenrequiring enzyme	HEM13	HEM13	F2QUX3
gene3637	Conserved predicted protein	PP7435_Chr3-0542	NA	F2QVS3
gene3748	Zinc-finger protein involved in transcriptional control of both nuclear and mitochondrial genes	ROP100	ROP100	F2QW29
gene4233	Serine threonine protein kinase	PP7435_Chr3-1110	NA	F2QXC5
gene4235	FAD/NAD(P)-binding domain	ERG1	ERG1	F2QXC7
gene4315	Dynactin subunit 1	PP7435_Chr3-1192	NA	F2QXK6
gene5370	Chorismate mutase, catalyzes the conversion of chorismate to prephenate	ARO7	ARO7	F2R0D7
gene5373	Peripheral membrane protein located at Vid (Vacuole import and degradation) vesicles	VID24	VID24	F2R0E0

300 genes downregulated in Only Δtrm1=300 genes; sector D

Id	Protein_name	Gene names (primary)	GI	UniRef100_Hit
gene5008	60S ribosomal protein L29	NA	NA	-
gene638	-	NA	NA	-
gene2526	-	NA	NA	-
gene2527	-	NA	NA	-
gene1082	-	NA	NA	-
gene4311	-	NA	NA	-
gene4188	-	NA	NA	-
gene1196	-	NA	NA	-
gene1197	-	NA	NA	-
gene1198	-	NA	NA	-
gene4310	-	NA	NA	-
gene4907	-	NA	NA	-

gene1725	-	NA	NA	-
gene880	-	NA	NA	-
gene2308	Predicted protein	NA	NA	-
gene1350	-	NA	NA	-
gene1351	-	NA	NA	-
gene4425	-	NA	NA	-
gene1824	-	NA	NA	-
gene3070	Predicted protein	NA	NA	-
gene1825	-	NA	NA	-
gene3190	-	NA	NA	-
gene3556	-	NA	NA	-
gene1827	-	NA	NA	-
gene1724	-	NA	NA	-
gene2032	-	NA	NA	-
gene2033	-	NA	NA	-
gene1777	-	NA	NA	-
gene1816	-	NA	NA	-
gene1826	-	NA	NA	-
gene498	-	NA	NA	-
gene1821	-	NA	NA	-
gene786	-	NA	NA	-
gene844	Mitochondrial genome maintenance protein MGM101	NA	NA	A0A1B2J5S2
gene2532	Similar to 40S ribosomal protein S14 acc.no.P19115	NA	NA	A0A1B2JBY6
gene1277	60S ribosomal protein L21-B	NA	NA	A0A1B2JE28
gene744	Conserved hypothetical membrane protein	PP7435_Chr1-2368	NA	A0A1G4KPB2
gene879	YVTN repeat-like/Quinoprotein amine dehydrogenase	ASC1	ASC1	A0A1G4KPC3
gene1195	60S ribosomal protein L18	RPL18B	RPL18B	A0A1G4KPF8
gene1372	Polycystic kidney disease protein 1-like 3	CCW14	CCW14	A0A1G4KPI2
gene1368	Predicted protein	RPS29A	RPS29A	A0A1G4KPI4
gene2368	Non-SMC subunit of the condensin complex(Smc2p-Smc4p-Ycs4p-Brn1p-Ycg1p)	YCG1	YCG1	A0A1G4KPV8
gene2444	U3 small nucleolar RNA-associated protein MPP10	MPP10	MPP10	A0A1G4KPW5

gene2531	40S ribosomal protein S22	RPS22B	RPS22B	A0A1G4KPY2
gene2854	3-hydroxy-3-methylglutaryl-CoA(HMG-CoA)synth ase, catalyzes the formation of HMG-CoA from acetyl-CoA	ERG13	ERG13	A0A1G4KQ20
gene3582	DNA-dependent RNA polymerase I subunit A43	RPA43	RPA43	A0A1G4KQB6
gene4041	Protein phosphatase 1 regulatory subunit 3	GAC1	GAC1	A0A1G4KQI9
gene4123	Glycolipid 2-alpha-mannosyltransferase 1	KTR1	KTR1	A0A1G4KQK0
gene4189	60S ribosomal protein L27	RPL27B	RPL27B	A0A1G4KQL4
gene4512	rRNA 2\'-O-methyltransferase fibrillarin	NOP1	NOP1	A0A1G4KQQ6
gene5234	Protein ECM3	PP7435_Chr4-0834	NA	A0A1G4KQX3
gene5200	ADP, ATP carrier protein 2	PP7435_Chr4-1818	NA	A0A1G4KQX5
gene5179	Nucleolar complex protein 2	NOC2	NOC2	A0A1G4KQY0
gene76	Enhancer of translation termination 1	NA	NA	C4QV62
gene81	DNA polymerase epsilon catalytic subunit A	NA	NA	C4QV66
gene551	Rpl2 60S ribosomal protein L2	NA	NA	C4QWG6
gene5310	Rpl2 60S ribosomal protein L2	NA	NA	C4QWG6
gene675	Protein required for partitioning of the 2-micro n plasmid	NA	NA	C4QWU2
gene1069	40S ribosomal protein S28	NA	NA	C4QXU7
gene1199	40S ribosomal protein S19	NA	NA	C4QY72
gene1264	Protein component of the large (60S) ribosomal subunit, has similarity to rat L38 ribosomal protein	NA	NA	C4QYD7
gene1282	Candida dubliniensis CD36 chromosome 4, complete sequence	NA	NA	C4QYF4
gene1330	40S ribosomal protein S0	NA	NA	C4QYK0
gene2943	alpha/beta-Hydrolases	NA	NA	C4QZN9
gene2832	Protein O-mannosyltransferase, transfers mannose residues from dolichylphosphate-D-mannose toprote	NA	NA	C4QZZ6
gene2771	YVTN repeat-like/Quinoprotein amine dehydrogenase	NA	NA	C4R055
gene2665	40S small subunit ribosomal protein S26A	NA	NA	C4R0F8
gene2307	U3 small nucleolar RNA-associated protein 5	NA	NA	C4R1F3
gene2205	Phosphoglucomutase, first 3 domains	NA	NA	C4R1Q2
gene2189	40S ribosomal protein S18	NA	NA	C4R1R7
gene1823	Glycolipid transfer protein (GLTP)	NA	NA	C4R2Q5
gene1802	U3 small nucleolar ribonucleoprotein protein LCP5	NA	NA	C4R2S2
gene1749	DNA-directed RNA polymerase I subunit RPA34.5 kDa polypeptide A34.5	NA	NA	C4R2X2
gene4087	Eukaryotic DNA topoisomerase I,N-terminal DNA-binding fragment	NA	NA	C4R3Z3

gene3990	60S ribosomal protein L43	NA	NA	C4R487
gene3873	Mitochondrial aspartate/glutamate carrier protein Aralar/Citrin (Contains EF-hand Ca2+-binding domains)	NA	NA	C4R4J8
gene3443	Mitochondrial group I intron splicing factor CCM1	NA	NA	C4R5P7
gene3302	Triosephosphate isomerase (TIM)	NA	NA	C4R626
gene3272	One of five related septins (Cdc3p, Cdc10p, Cdc11p, Cdc12p, Shs1p)	NA	NA	C4R655
gene5197	Ribose-5-phosphate isomerase	NA	NA	C4R764
gene4984	Inheritance of peroxisomes protein 1	NA	NA	C4R7R4
gene4831	40S ribosomal protein S1	NA	NA	C4R853
gene4525	H/ACA ribonucleoprotein complex subunit 2	NA	NA	C4R8Z0
gene27	S-adenosyl-L-methionine-dependent methyltransferases	HMT1	HMT1	F2QLC0
gene1083	Acetyl-CoA carboxylase, biotin containing enzyme	ACC1	ACC1	F2QLC7
gene1097	Ribosome biogenesis protein ERB1	ERB1	ERB1	F2QLE1
gene1110	60S ribosomal protein L33-A	RPL33A	RPL33A	F2QLF3
gene1123	Nucleolar protein required for the normal accumulation of 25S and 5.8S rRNA	URB1	URB1	F2QLG6
gene1130	Phosphoglycerate kinase	PGK1	PGK1	F2QLH3
gene1133	Fatty acid synthase subunit alpha	FAS2	FAS2	F2QLH6
gene989	Abnormal spindle-like microcephaly-associated protein	GZF3	GZF3	F2QLI0
gene1033	Midasin	SRP40	SRP40	F2QLM3
gene1073	Protein component of the large (60S) ribosomal subunit, nearly identical to Rpl34Bp	RPL34A	RPL34A	F2QLQ8
gene28	Protein component of the large (60S) ribosomal subunit, nearly identical to Rpl4Ap	RPL4A	RPL4A	F2QLR1
gene47	FK506-binding protein 15	PP7435_Chr1-0049	NA	F2QLT0
gene63	60S acidic ribosomal protein P0	RPP0	RPP0	F2QLU5
gene117	RNA-binding domain, RBD	NOP6	NOP6	F2QLZ3
gene121	40S ribosomal protein S3	RPS3	RPS3	F2QLZ7
gene184	Subunit beta1 of the nascent polypeptide-associated complex (NAC) involved in protein targeting	EGD1	EGD1	F2QM52
gene369	GTPase that associates with nuclear 60S pre-ribosomes, required for export of 60S ribosomal subunits	NUG1	NUG1	F2QM69
gene378	U3 small nucleolar RNA-associated protein 8	UTP8	UTP8	F2QM77
gene394	40S ribosomal protein S22	RPS22A	RPS22A	F2QM88
gene408	Rrs1 ribosome biogenesis and nuclear export protein	RRS1	RRS1	F2QMA0
gene487	N-glycosylated protein involved in the maintenance of bud site selection during bipolar budding	RAX2	RAX2	F2QMH6
gene497	Negative regulator of theglucose-sensing signal transduction pathway	MTH1	MTH1	F2QMI6

gene499	NB8M (B18) subunit ofmitochondrialNADH:ubiquinone oxidoreductase (Complex I)	NB8M	NB8M	F2QMI7
gene511	60S ribosomal protein L1-B	RPL1B	RPL1B	F2QMJ7
gene518	60S ribosomal protein L12	RPL12B	RPL12B	F2QMK4
gene530	Type I PLP-dependent aspartate aminotransferase-like (Major domain)	AAT1	AAT1	F2QML6
gene700	Protein involved in mRNA turnover and ribosome assembly, localizes to the nucleolus	MRT4	MRT4	F2QMM2
gene707	Conserved predicted protein	PP7435_Chr1-0691	NA	F2QMM9
gene721	Protein component of the large (60S) ribosomal subunit, nearly identical to Rpl9Ap	RPL9B	RPL9B	F2QMN9
gene779	Ilv5 ketol-acid reductoisomerase	ILV5	ILV5	F2QMT7
gene787	40S ribosomal protein S8	RPS8A	RPS8A	F2QMU3
gene958	Essential helicase component of hetero hexameric MCM2-7 complexes	MCM4	MCM4	F2QN89
gene240	Essential protein involved in rRNA and snoRNA maturation	PXR1	PXR1	F2QNG0
gene328	Essential conserved nucleolar protein necessary for biogenesis of 60S ribosomal subunits	RRP1	RRP1	F2QNP3
gene548	Ribosomal protein L4 of the large(60S)ribosomal subunit, nearly identical to Rpl8Ap	RPL8B	RPL8B	F2QNT9
gene566	Protein component of the large (60S) ribosomal subunit, nearly identical to Rpl9Ap	RPL9A	RPL9A	F2QNV7
gene639	60S ribosomal protein L35	RPL35B	RPL35B	F2QP29
gene650	Homocitrate synthase isozyme, catalyzes the condensation of acetyl-CoA and alpha-ketoglutarate	LYS21	LYS21	F2QP40
gene674	Ribosomal protein L37	RPL37A	RPL37A	F2QP63
gene678	Transferase(Phosphotransferase) domain 1	KCC4	KCC4	F2QP65
gene681	60S ribosomal protein L5-B	RPL5	RPL5	F2QP67
gene687	Protein SLF1	SRO9	SRO9	F2QP72
gene696	Acyl-CoA N-acyltransferases (Nat)	PP7435_Chr1-0678	NA	F2QP81
gene1139	Mitochondrial external NADH dehydrogenase, a type II NAD(P)H:quinone oxidoreductase	NDE1	NDE1	F2QP86
gene1165	DNA-directed RNA polymerase	RPA135	RPA135	F2QPB2
gene1178	Conserved nuclear RNA-binding protein	THO1	THO1	F2QPC3
gene1205	Likely mitochondrial ribosomal protein (E.coli L7/L12)	MNP1	MNP1	F2QPE5
gene1206	DNA-directed RNA polymerase subunit	RPB9	RPB9	F2QPE6
gene1221	Genomic scaffold, Kuraishia_capsulata_scaffold_4	PP7435_Chr1-1159	NA	F2QPG1
gene1244	Transcriptional activator of genes regulated by nitrogen catabolite repression (NCR)	FEP1	FEP1	F2QPI3
gene1276	40S ribosomal protein S9-A	RPS9B	RPS9B	F2QPL3
gene1284	Isopentenyl diphosphate:dimethylallyldiphosph ate isomerase (IPPisomerase)	IDI1	IDI1	F2QPM0
gene1352	40S ribosomal protein S15	RPS15	RPS15	F2QPT5

gene1354	RNA-binding domain, RBD	NOP12	NOP12	F2QPT7
gene1392	Subunit of the heterodimeric FACT complex(Spt16p-Pob3p)	SPT16	SPT16	F2QPX0
gene1420	P-loop containing nucleosidetriphosphatehydrolases	RFC5	RFC5	F2QPZ9
gene1435	Sphingolipid long chainbase-responsive protein PIL1	PIL1	PIL1	F2QQ12
gene1436	High-affinity glucose transporter of the major facilitator superfamily	PpHXT1	PpHXT1	F2QQ13
gene1460	40S ribosomal protein S2	RPS2	RPS2	F2QQ33
gene1516	YVTN repeat-like/Quinoprotein amine dehydrogenase	UTP15	UTP15	F2QQ87
gene1533	Proteasome-interacting protein CIC1	CIC1	CIC1	F2QQA2
gene1582	60S ribosomal protein L36	RPL36A	RPL36A	F2QQF1
gene1586	Lactose regulatory protein LAC9 and GAL4-likeprotein	GAL4	GAL4	F2QQF5
gene1600	P-loop containing nucleosidetriphosphatehydrolases	TEF2	TEF2	F2QQG8
gene1702	U3 small nucleolar RNA-associated protein 10(U3 snoRNA-associated protein 10)	UTP10	UTP10	F2QQR5
gene1718	Nucleoside diphosphate kinase, NDK	YNK1	YNK1	F2QQT0
gene1722	Transmembrane protein 165	PP7435_Chr2-0066	NA	F2QQT5
gene1753	30S ribosomal protein S5	MRPS5	MRPS5	F2QQW2
gene1766	Essential protein required for the maturation of 25S rRNA and 60S ribosomal subunit assembly	EBP2	EBP2	F2QQX3
gene1776	60S ribosomal protein L17	RPL17B	RPL17B	F2QQY3
gene1785	Glutathione S-transferase, C-terminal domain	GTT1	GTT1	F2QQZ1
gene1801	Ribosome biogenesis protein nsa2	NSA2	NSA2	F2QR04
gene1815	Bifunctional carbamoylphosphatesynthetase(CPSase)-aspartate transcarbamylase (ATCase)	URA2	URA2	F2QR18
gene1828	Pseudouridine synthase catalytic subunit of box H/ACA small nucleolar ribonucleoprotein particles	CBF5	CBF5	F2QR23
gene1834	Muscle M-line assembly protein unc-89	PP7435_Chr2-0160	NA	F2QR29
gene1847	C-terminal domain of alpha and beta subunits of F1 ATP synthase	ATP2	ATP2	F2QR41
gene1896	Serine/threonine-protein kinase MRCK beta	PP7435_Chr2-0216	NA	F2QR85
gene1921	FKBP-like	FPR3	FPR3	F2QRA9
gene1952	30S ribosomal protein S10	RPS20	RPS20	F2QRD7
gene1953	Endosomal integral membrane protein	PP7435_Chr2-0271	NA	F2QRD8
gene2011	Subunit of the multiprotein cohesin complex required for sister chromatid cohesion in mitotic cells	SMC3	SMC3	F2QRJ1
gene2034	40S ribosomal protein S25	RPS25A	RPS25A	F2QRL2
gene2183	Cupredoxins - blue copper proteins	FET3	FET3	F2QRZ2
gene2194	40S ribosomal protein S17 subunit	RPS17B	RPS17B	F2QS01

gene2208	Pyruvate kinase	CDC19	CDC19	F2QS13
gene2255	60S ribosomal protein L28	RPL28	RPL28	F2QS58
gene2256	HD domain-containing protein 2	PP7435_Chr2-0550	NA	F2QS59
gene2292	Acyltransferase that catalyzes diacyl glycerol esterification	RIX1	RIX1	F2QS95
gene2330	Beta-1,3-glucan synthase catalytic subunit1	FKS1	FKS1	F2QSC9
gene2333	Ribosomal protein L15	RPL15B	RPL15B	F2QSD2
gene2359	40S ribosomal protein S27	RPS27B	RPS27B	F2QSF5
gene2369	Glucosamine--fructose-6-phosphate aminotransf erase (Isomerizing)	GFA1	GFA1	F2QSG4
gene2394	YVTN repeat-like/Quinoprotein amine dehydrogenase	DIP2	DIP2	F2QSI7
gene2418	40S ribosomal protein S11	RPS11B	RPS11B	F2QSL1
gene2426	Diphthine synthase	DPH5	DPH5	F2QSL9
gene2452	Deoxyhypusine hydroxylase	LIA1	LIA1	F2QSP4
gene2484	Translationally controlled tumor protein homolog (TCTP)	TMA19	TMA19	F2QSS6
gene2528	Phosphatidylinositol 3-kinase Catalytic Subunit| Chain A| domain 1	RPL40A	RPL40A	F2QSW4
gene2529	Genomic scaffold, Kuraishia_capsulata_scaffold	PP7435_Chr2-0810	NA	F2QSW5
gene2547	HSP-interacting, SSA1 ATPaseactivity-stimulating, TPR-containing co-chaperone	CNS1	CNS1	F2QSY1
gene2649	Translation elongation factor 1-beta(EF-1-beta)	EFB1	EFB1	F2QT76
gene2678	Transcription elongation factor SPT4	SPT4	SPT4	F2QTA2
gene2720	NAD(P)-binding Rossmann-fold domains	PP7435_Chr2-0990	NA	F2QTE2
gene2785	Phosphatidylinositol4,5-bisphosphate-binding protein SLM1	PP7435_Chr2-1051	NA	F2QTK0
gene2820	U3 snoRNP protein, component of the small(Ribosomal) subunit (SSU) processosome containing U3snoRN	NAN1	NAN1	F2QTN2
gene2843	S-adenosylmethionine decarboxylase proenzyme	SPE2	SPE2	F2QTQ4
gene2859	Tubulin alpha chain	TUB1	TUB1	F2QTR9
gene2881	Protein that is processed in the mitochondrion to yield acetylglutamate kinase and N-acetyl-gamma-gl	ARG5,6	ARG5,6	F2QTU0
gene2890	Cobalamin-independent methioninesynthase, involved in amino acid biosynthesis	MET6	MET6	F2QTU9
gene2959	RNA-binding domain, RBD	PAB1	PAB1	F2QU09
gene2960	P-loop containing nucleosidetriphosphatehydrolases	RLI1	RLI1	F2QU10
gene2963	DNA mismatch repair protein MSH6	MSH6	MSH6	F2QU13
gene2971	Protein component of the large (60S) ribosomal subunit, has similarity to rat L30 ribosomal protein	RPL30	RPL30	F2QU20
gene2972	Ribosomal protein L30 of the large (60S) ribosom al subunit, nearly identical to Rpl24Ap	RPL24B	RPL24B	F2QU21
gene2980	Plasma membrane G protein coupled receptor(GPCR) that interacts with the heterotrimeric G protein	GPR1	GPR1	F2QU29

gene2994	Conserved predicted protein	PP7435_Chr2-1246	NA	F2QU43
gene3038	Protein component of the large (60S) ribosomal subunit, nearly identical to Rpl7Ap	RPL7A	RPL7A	F2QU82
gene3116	mannose-6-phosphate isomerase, class I	PMI40	PMI40	F2QUE3
gene3171	Exosome complex protein LRP1	LRP1	LRP1	F2QUI9
gene3189	Protein component of the large (60S)ribosomal subunit, has similarity to rat L32 ribosomal protein	RPL32	RPL32	F2QUK5
gene3205	13 kDa ribonucleoprotein-associated protein	SNU13	SNU13	F2QUM0
gene3209	6,7-dimethyl-8-ribityllumazine synthase	RIB4	RIB4	F2QUM4
gene3215	Essential nuclear protein involved in proteasome maturation and synthesis of 40S ribosomal subunits	NOB1	NOB1	F2QUN0
gene3224	YVTN repeat-like/Quinoprotein amine dehydrogenase	PWP1	PWP1	F2QUN8
gene3292	Replication factor A protein 3	PP7435_Chr3-0210	NA	F2QUV4
gene3296	Cytochrome P-450 lanosterol 14-alpha-demethylase	ERG11	ERG11	F2QUV8
gene3349	54S ribosomal protein L3	MRPL3	MRPL3	F2QV04
gene3355	Mitochondrial peroxiredoxin (1-Cys Prx) with thioredoxin peroxidase activity	PRX1-1	PRX1-1	F2QV08
gene3373	Adenosylhomocysteinase	SAH1	SAH1	F2QV27
gene3377	Nucleolar protein, component of the small subunit (SSU) processome containing the U3 snoRNA	UTP5	UTP5	F2QV31
gene3388	S-adenosylmethionine synthase	SAM2	SAM2	F2QV42
gene3401	Acid proteases	YPS1-5	YPS1-5	F2QV55
gene3437	Conserved predicted protein	PP7435_Chr3-0348	NA	F2QV88
gene3497	YVTN repeat-like/Quinoprotein amine dehydrogenase	UTP13	UTP13	F2QVE4
gene3522	40S ribosomal protein S5	RPS5	RPS5	F2QVG5
gene3557	Heat shock protein of the HSP70 family (SSB1) (HSP75)	SSB2	SSB2	F2QVJ7
gene3566	Phosphatidylinositol 3-kinase Catalytic Subunit| Chain A| domain 1	RPS31	RPS31	F2QVK8
gene3590	Eukaryotic translation initiation factor 3 subunit B	PRT1	PRT1	F2QVN1
gene3619	D-lactate dehydrogenase	DLD2	DLD2	F2QVQ6
gene3623	Thioredoxin-like	TRX2	TRX2	F2QVR0
gene3630	Cytochrome B pre-mRNA-processing protein 6	CBP6	CBP6	F2QVR7
gene3632	Siderophore iron transporter 1	SIT1-1	SIT1-1	F2QVR9
gene3643	NAP family histone chaperone	VPS75	VPS75	F2QVS9
gene3644	DNA-directed RNA polymerase I subunit A49	RPA49	RPA49	F2QVT0
gene3675	Aspartate/ornithine carbamoyl transferase, Asp/ Orn binding domain	ARG3	ARG3	F2QVV9
gene3691	Similar to S. cerevisiae YNL190W	PP7435_Chr3-0593	NA	F2QVX3

gene3701	Elongation of fatty acids protein	ELO2	ELO2	F2QVY3
gene3707	40S ribosomal protein S21	RPS21A	RPS21A	F2QVY9
gene3708	P-loop containing nucleosidetriphosphatehydrolases	TIF2	TIF2	F2QVZ0
gene3716	40S ribosomal protein S12	RPS12	RPS12	F2QVZ9
gene3717	Rab proteinsgeranylgeranyltransferase component A	MRS6	MRS6	F2QW00
gene3730	Component of the small (Ribosomal) subunit(SSU) processosome required for pre-18S rRNA processing	SAS10	SAS10	F2QW12
gene3850	Glucose-6-phosphate isomerase like protein|domain 1	PGI1	PGI1	F2QWD0
gene3892	Lyase| Ornithine Decarboxylase| ChainA|domain 1	SPE1	SPE1	F2QWH1
gene3930	Mitochondrial import inner membrane translocase subunit TIM9	TIM9	TIM9	F2QWK7
gene3983	Eukaryotic translation initiation factor 3 subunit A	RPG1	RPG1	F2QWP9
gene4002	DNA topoisomerase 2	TOP2	TOP2	F2QWR7
gene4034	Essential protein possibly involved in secretion	BFR2	BFR2	F2QWU8
gene4097	proliferating cell nuclear antigen (pcna)	POL30	POL30	F2QX08
gene4121	Arg1 argininosuccinate synthase	ARG1	ARG1	F2QX29
gene4127	tRNA (Uracil-O(2)-)-methyltransferase	TRM44	TRM44	F2QX34
gene4148	Similar to 60S ribosomal protein L44 acc.no.Q9UVB8	RPL42B	RPL42B	F2QX54
gene4193	Likely nucleolar ribosomal biogenesis factor BRX1p	BRX1	BRX1	F2QX91
gene4260	60S acidic ribosomal protein P2	RPP2A	RPP2A	F2QXF2
gene4274	Phosphoribosylaminoimidazolecarboxylase, cata lyzes a step in the \'de novo\' purinenucleotide biosynthesis	ADE2	ADE2	F2QXG6
gene4327	Mitochondrial 2-oxodicarboxylate carrier 2	ODC1	ODC1	F2QXL8
gene4424	40S ribosomal protein S10	RPS10A	RPS10A	F2QXR5
gene4423	40S ribosomal protein S6	RPS6B	RPS6B	F2QXR6
gene4376	ATP synthase E chain	TIM11	TIM11	F2QXW1
gene4375	DNA helicase and DNA replication licensing factor	MCM7	MCM7	F2QXW2
gene4504	Primary rRNA-binding ribosomal protein component of the large (60S) ribosomal subunit	RPL25	RPL25	F2QY34
gene4517	Ribosome production factor 1	RPF1	RPF1	F2QY44
gene4522	54S ribosomal protein L7	MRPL7	MRPL7	F2QY49
gene4548	YVTN repeat-like/Quinoprotein amine dehydrogenase	ELP2	ELP2	F2QY76
gene4607	N-terminal domain of eukaryotic peptide chain release factor subunit 1, ERF1	SUP45	SUP45	F2QYD2
gene4619	Pescadillo	NOP7	NOP7	F2QYE4
gene4651	60S acidic ribosomal protein P1	RPP1B	RPP1B	F2QYH3

gene4672	Protein component of the large (60S) ribosomal subunit, nearly identical to Rpl11Bp	RPL11B	RPL11B	F2QYJ3
gene4761	Acid proteases	YPS1-1	YPS1-1	F2QYS7
gene4767	Carbonic anhydrase	NCE103	NCE103	F2QYT3
gene4795	Zinc metalloprotease zmpB	PP7435_Chr4-0421	NA	F2QYW0
gene4818	Permease of basic amino acids in the vacuolar membrane	VBA1-2	VBA1-2	F2QYY1
gene4832	Inosine-5\'-monophosphate dehydrogenase	IMD3	IMD3	F2QYZ3
gene4843	Nucleolar protein, forms a complex with Noc4p	NOP14	NOP14	F2QZ04
gene4870	Exonuclease 1	EXO1	EXO1	F2QZ29
gene4879	Ribosomal protein lysine methyltransferase	RKM2	RKM2	F2QZ37
gene4897	AdoMet-dependent rRNA methyltransferase SPB1	SPB1	SPB1	F2QZ52
gene4906	40S ribosomal protein S13	RPS13	RPS13	F2QZ60
gene4957	40S ribosomal protein S16	RPS16B	RPS16B	F2QZA5
gene4958	60S ribosomal protein L13	RPL13B	RPL13B	F2QZA6
gene4967	Serine/threonine-protein kinase has pin homolog hrk1	ALK2	ALK2	F2QZB5
gene5002	Cytochrome b5-like Heme/Steroid binding domain	CYB5-1	CYB5-1	F2QZE5
gene5036	40S ribosomal protein S23	RPS23A	RPS23A	F2QZH5
gene5101	P-loop containing nucleosidetriphosphatehydrolases	CDC12	CDC12	F2QZN5
gene5102	40S ribosomal protein S24	RPS24A	RPS24A	F2QZN6
gene5122	Nucleolar protein 58	NOP58	NOP58	F2QZQ1
gene5127	Essential nucleolar protein involved in the early steps of 35S rRNA processing	FCF2	FCF2	F2QZQ6
gene5134	60S ribosomal protein L26-B	RPL26B	RPL26B	F2QZR3
gene5158	40S ribosomal protein S4	RPS4B	RPS4B	F2QZT6
gene5162	Fe II, 2-oxoglutarate-dependent dioxygenase	TPA1	TPA1	F2QZU0
gene5199	Protein component of the small (40S) ribosomal subunit, nearly identical to Rps7Bp	RPS7B	RPS7B	F2QZX4
gene5201	Major ADP/ATP carrier of the mitochondrial inner membrane	PET9	PET9	F2QZX5
gene5202	N-terminally acetylated protein component of the large (60S) ribosomal subunit, binds to 5.8 S rRNA	RPL16A	RPL16A	F2QZX6
gene5222	Cyclin-like protein that interacts with Pho85p	CLG1	CLG1	F2QZZ7
gene5231	60S ribosomal protein L31	RPL31B	RPL31B	F2R006
gene5232	Potential tRNA dihydrouridinesynthase similar to S. cerevisiae DUS4 (YLR405W)	DUS4	DUS4	F2R007
gene5233	tRNA-dihydrouridine (47) synthase	DUS3	DUS3	F2R008
gene5244	DNA binding apoptosis-related protein	SDD2	SDD2	F2R021

gene5278	60S ribosomal protein L3	RPL3	RPL3	F2R050
gene5286	60S ribosomal protein L6	RPL6B	RPL6B	F2R058
gene5290	RNAse III, putative (Double-strandedribonuclease, putative)	RNT1	RNT1	F2R062
gene5312	Nucleolar GTP-binding protein 1 (NOG1)	NOG1	NOG1	F2R084
gene5323	40S ribosomal protein S6	RPS6A	RPS6A	F2R095
gene5327	S-adenosyl-L-methionine-dependent methyltrans ferases	TGS1	TGS1	F2R099
gene5368	Omega-6 fatty acid desaturase (Delta-12 desaturase)	ODE1	ODE1	F2R0D5
gene5384	P-loop containing nucleosidetriphosphatehydrolases	YEF3	YEF3	F2R0F1
gene2623	ATP-dependent 6-phosphofructokinase subunit alpha	NA	NA	#NAME?
