# Supplementary material for: Coordinate regulation of methanol utilization pathway genes of Komagataella phaffii by transcription factors and chromatin modifiers
Source: Front Microbiol. 2022 Sep 6;13:991192. doi: 10.3389/fmicb.2022.991192 (PMC9485576; doi:10.3389/fmicb.2022.991192)
Supplement: Supplementary file 2 [file Data_Sheet_2.PDF]

A.

UniRef	Gene	log2FoldChange (*Δgcn5*)
F2QZI8	PP7435_Chr4-0658	-4.114522087
F2R0E0	VID24	-2.338215458
F2QMS4	ADY2-2	-2.118112957
C4R0F6	-	-2.063053993
F2QZY0	TFB3	-1.986147058
F2QLS8	BIO2	-1.949401165
F2QM33	UIP4	-1.944370768
F2QNT1	PP7435_Chr1-0522	-1.744954577
F2QPA2	ERG20	-1.557087151

B.

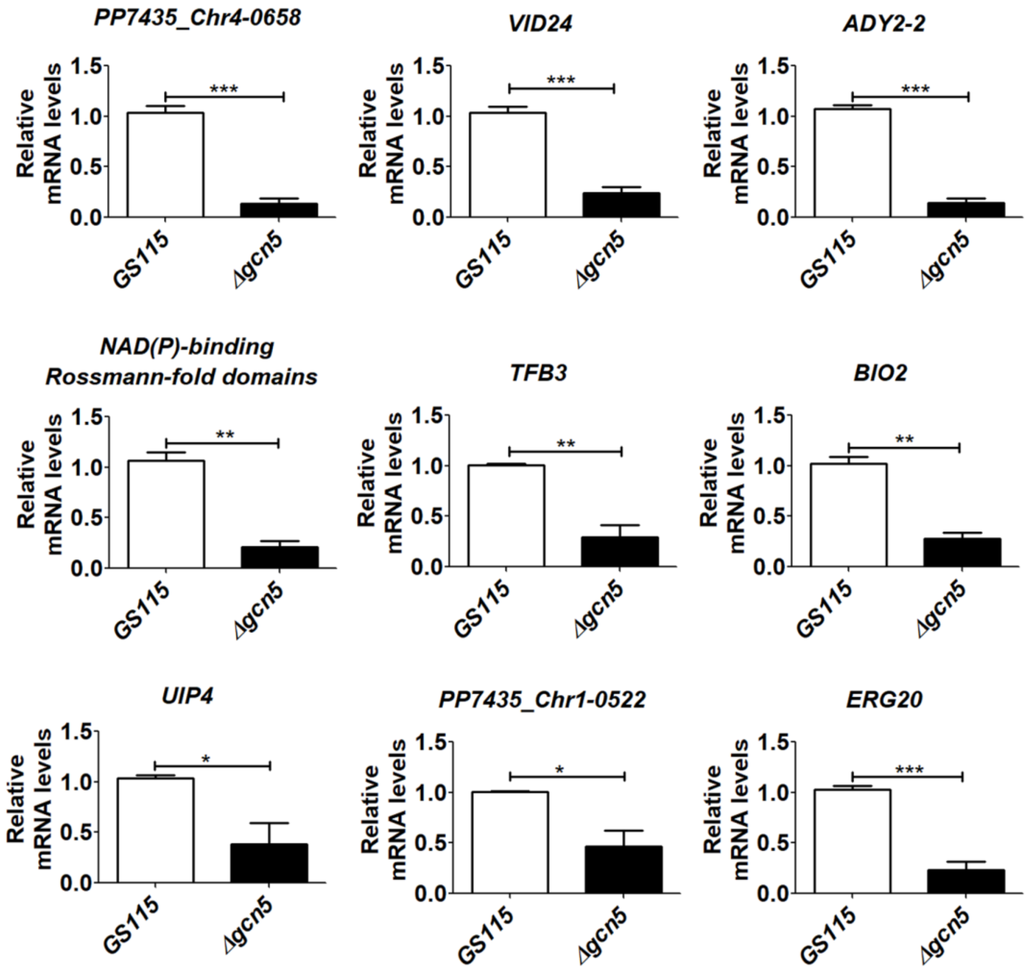

A. Genes which are downregulated from 4.1 to 1.5 fold in *Δgcn5* as evident from RNA seq analysis (Supporting information 1) were chosen randomly for qPCR validation. **B.** Analysis of mRNA levels of select genes downregulated in *Δgcn5*. Cells were cultured in cultured in YPM for 6 h. Error bars in each figure indicate S.D. n=3.
